# Assessing psychotherapy patients’ social context through personal networks and experience sampling: A mixed methods case series exploring feasibility

**DOI:** 10.1016/j.nsa.2026.107029

**Published:** 2026-09-09

**Authors:** Marie Stadel, Janneke van Houts, Shanice Wortel, Barbara Montagne, Laura F. Bringmann

**Affiliations:** a Department of Methodology and Statistics, Tilburg University, Tilburg, the Netherlands; b Department of Sociology, University of Groningen, Groningen, the Netherlands; c Department of Psychometrics and Statistics, University of Groningen, Groningen, the Netherlands; d Tilburg Experience Sampling Center, Tilburg University, Tilburg, the Netherlands; e GGz Central, Amersfoort, the Netherlands; f Department of Psychology, Utrecht University, Utrecht, the Netherlands

**Keywords:** Ecological momentary assessment, Ambulatory assessment, Sociogram, Social context, Digital mental health, Clinical feasibility

## Abstract

This study explored the feasibility and perceived clinical utility of a novel psychotherapy intervention consisting of two components (1) assessment of patients' social context using the experience sampling methodology (ESM) and personal social network data collection (PSN) and (2) a feedback report summarizing the collected data. Four qualitatively driven concurrent embedded mixed-methods case studies were conducted in a large mental health care facility in the Netherlands and included nine young adults receiving either individual or group psychotherapy: six in part-time treatment and three transitioning from inpatient to outpatient care. Feasibility and utility were evaluated using codebook thematic analysis of semi-structured patient and therapist interviews, patient compliance with the intervention components and researchers' reflexive field notes. Overall, the intervention appeared most feasible in individual therapy, in which patient and therapist engaged with it collaboratively. Engagement was more difficult among avoidant patients and when the intervention was experienced as *too confronting* by patients, highlighting the importance of establishing rapport before its use. The intervention yielded varying degrees of insight for patients, depending on their ability to engage with the assessments and feedback-based therapeutic dialogue. The feedback report specifically improved therapists' understanding of patients’ social context. Future implementation will require automation, integration into digital clinical infrastructure, and therapist training to support appropriate interpretation of feedback and follow-up action.

## 1 Introduction

Understanding a person's daily social life is important for understanding their mental health. Social context can improve or aggravate wellbeing (Bertera, 2005; Yanos et al., 2001; Southwick et al., 2016). For example, social support can help in buffering for, dealing with, and recovering from existing psychopathology (Brown et al., 2011; Scardera et al., 2020), while adverse social interactions or the absence of social contact can have deteriorating effects on mental health (Bertera, 2005; Yanos et al., 2001; Lakey and Cronin, 2008; Rook, 1984). A recent meta-analysis of the effectiveness of social network interventions for psychiatric patients indicates that improving social functioning also leads to improvements in psychopathological symptoms and better treatment adherence (Swinkels et al., 2023). Social functioning and a patient's social network are therefore important transdiagnostic factors to be considered in psychotherapy (McEvoy et al., 2013; Girard et al., 2017; Leijdesdorff et al., 2022).

Several evidence-based psychotherapeutic approaches emphasize the importance of patients' social functioning or explicitly incorporate social functioning in treatment, depending on the approach (see Table 1 for a brief overview). However, only little attention has been paid to *systematically* assessing and using information about a patient's daily social life in clinical practice (Batstra and Frances, 2025; Kinderman et al., 2020; Wakefield and First, 2012). While daily-life situations and interpersonal experiences are frequently addressed in sessions, patients' reports are likely to be selective, with recall and disclosure biased toward more recent or emotionally salient situations (Ryan and Kahn, 2015; Stull et al., 2009; Holland and Kensinger, 2010). Psychotherapy may therefore benefit from a structured overview of patients' broader social environments (Stadel et al., 2023). Connected to that, several studies with different patient groups indicate that clinical care-as-usual has little success in improving patients' social functioning (Devoe et al., 2019; Gunderson et al., 2011; Renner et al., 2014; Zanarini et al., 2010). This is concerning, as persistent social difficulties are associated with increased risk of relapse (Leijdesdorff et al., 2022). It may thus be useful to capture and examine patients' social context in a more detailed and structured way than currently done in clinical practice.

**Table 1:** Overview of evidence-based psychotherapy approaches’ use of social context.

Psychotherapy Approach	Use of Social Context	Reference
Acceptance and Commitment Therapy (ACT)	Social life not at the center, rather focused on personal resilience and values; values often include connection, which relates to social behavior	Hayes et al. (2011)
Cognitive Behavioral Analysis System of Psychotherapy (CBASP)	Interpersonal disconnection is the core problem related to depression; in situational analyses, interpersonal interactions are discussed in therapy; improvement occurs through changing interpersonal behavior	McCullough (2003)
Cognitive Behavioral Therapy (CBT)	Social functioning provides context for symptoms; therapy often targets thoughts/behaviors in daily social situations	Beck (1979)
Dialectical Behavior Therapy (DBT)	Developing interpersonal effectiveness skills and improving relationship stability is at the core of this treatment	Linehan (1993)
Emotion-Focused Therapy (EFT)	Focus on emotions in the context of social relationships (e.g., in couples therapy); emotion regulation in interpersonal contexts as main treatment target	Klerman and Weissman (1994)
Interpersonal Psychotherapy (IPT)	Social life of the patients is the primary focus (e.g., interpersonal conflict or role transitions); social relationships are explored in a structured way; changing interpersonal functioning is central to improvement	Greenberg (2004)
Mentalization-Based Therapy (MBT)	Focus on understanding self/others in relationships, particularly relational misunderstandings	Bateman and Fonagy (2004)
Mindfulness-based cognitive therapy (MBCT)	Focuses mainly on internal awareness rather than interpersonal behavior, but includes cultivating compassion and mindfulness in relating to others	Segal et al. (2012)
Psychodynamic Psychotherapy	Interpersonal patterns from early relationships are explored; focus on transference (i.e., unconscious feeling/beliefs about past relationships are redirected to present relationships)	Lemma (2024)
Schema Therapy	Relationships in the present trigger (maladaptive) schemas (rooted in past relationships); improvement occurs through changing interpersonal patterns	Young et al. (2006)

A new promising approach to do so is combining personal social networks (PSN) and experience sampling methodology (ESM) (Stadel et al., 2023, 2024). A PSN maps out a patient's social connections – who is part of their social network and what kind of relationship does the patient have with these people. A recent study by Nicaise and colleagues (Nicaise et al., 2022) shows that patients and clinicians perceived feedback about a patient's PSN as useful for treatment, and patients reported increased satisfaction with their social support resources. In addition to a PSN, ESM can be used to gain insight into a patient's daily social activity and its relation to symptoms. By delivering multiple brief questionnaires to the patient's smartphone throughout everyday life ESM captures experiences in real time and in naturalistic contexts (see e.g., Brown et al., 2011). This allows for the collection of ecologically valid data while minimizing recall bias. As a result, ESM has become increasingly popular in psychotherapy research, particularly because it may support more individualized assessment and treatment approaches (aan het Rot et al., 2012; Myin-Germeys et al., 2009; Trull and Ebner-Priemer, 2009).

When combining PSN and ESM it becomes possible to assess with which members of their social network patients interact, how these specific interactions are experienced, and how they may relate to fluctuations in symptoms (Stadel et al., 2024). Like with other clinical ESM implementations (e.g., Bringmann et al., 2021; Klipstein et al., 2023), the data collected through PSN and ESM can be summarized into an interactive feedback report to be reviewed jointly by patient and therapist (Stadel et al., 2023). Such shared exploration may support a more nuanced understanding of the patient's difficulties, help identify new treatment targets, and provide a diverse set of concrete daily-life situations to discuss in therapy (Bos, 2021).

To explore the clinical utility of a feedback report summarizing patients' social context, Stadel and colleagues (Stadel et al., 2023) conducted a focus group discussion during which psychotherapy patients were asked to reflect on the potential of the integration of ESM and PSN. The patients considered the use of ESM and PSN valuable but did not expect to gain new insights themselves from a feedback report summarizing their social life. Instead, patients expected that the report would be useful for communication with their therapist, and that it would help the therapist to understand the patient's complaints better. However, to date, the combination of PSN with ESM and associated feedback has not been tested in clinical practice yet, and no information on the clinician perspective is available.

Therefore, in this study we piloted the application of the integration of PSN and ESM as an intervention within a clinical setting where different patients complete the assessment and feedback procedure accompanied by their therapists. Concretely, we conducted a series of four exploratory, mixed-methods case studies with young adults with severe mental health problems in transdiagnostic treatment within one large mental healthcare facility in the Netherlands. Thereby, we were interested in the patient and the therapist perspective on the utility and feasibility of the intervention.

## 2 Methods

### 2.1 Design & ethics

We used a concurrent embedded mixed methods multiple-case study design. The primary strand was qualitative, focusing on how the intervention was experienced by patients, therapists and researchers. Quantitative data were used as a supporting strand to describe compliance and data characteristics of obtained ESM and PSN data.

The study protocol was assessed by the Medical Ethical Committee at the University Medical Centre Groningen (UMCG) and judged as not requiring full review as we asked little involvement of patients beyond standard care (METc, 2023/285). To guarantee secure data storage, all research activities took place within the mental healthcare facility and its digital environment. We aim to report our research study as transparently as possible while protecting participants' anonymity. In preparing this manuscript, we followed the Standards for Reporting Qualitative Research (SRQR; O'Brien et al., 2014). Because of space constraints, information on researcher characteristics, the study's research paradigm, and a summary of the strategies to enhance trustworthiness are provided in the supplementary materials.

### 2.2 Case selection

The study took place in 2025 within two departments of the mental healthcare facility, both specializing in the treatment of severe mental health complaints transdiagnostically. One focuses on young adults, and the other is explicitly transdiagnostic, treating a broader clinical group. To compose our sample, we used a purposive sampling approach (Patton, 2014) focusing on patients who struggle with social functioning according to their psychotherapists as this would deliver the richest data for our study aim. Within this group, we deliberately sampled patients with different diagnoses at different stages of treatment to understand when our intervention might be most useful. Because the consulted therapists, who served as gatekeepers, considered group applications of the intervention particularly useful, we also allowed for the inclusion of groups. Our final sample included, across four case studies, a total of nine young adults (median age = 23.5, age range = 20 to 29, 67% female) and their treating psychotherapists. This included two group cases, one with four and one with three patients (*Case Studies 1 & 2,* respectively), and two individual therapy cases (*Case Studies 3 & 4*). Table 2 provides an overview of the patients’ demographic information and diagnoses.

**Table 2:** Demographic information of participants.

Patient	Case Study	Gender	Age	In treatment for
001	1	Female	28	Post-Traumatic Stress Disorder
002		Male	24	Social Anxiety Disorder
003		Female	?	?
004		Female	23	Avoidance
005	2	Male	23	Obsessive Compulsive Disorder
006		Female	29	Borderline and Post-Traumatic Stress Disorder
007		Female	29	Obsessive Compulsive Disorder
008	3	Female	23	Anxiety and Eating Disorder
009	4	Male	20	Panic Disorder, Attachment Problems and Personality Disorder

*Note.* Participant 003 dropped out of the study after two days of the ESM period. We did not obtain demographic information from this participant as this was assessed during the evaluation interview.

#### 2.2.1 Case study 1 (CS1): part-time group therapy

As a first case study we could integrate our research procedure into a part-time treatment program for young adults aged between 17 and 25. Patients in this program have different diagnoses, but all suffer from problems at work/school and in their social environment, which lead to stagnation in their lives and irregular day structures. Patients spend two full days per week at the mental healthcare facility during which they work on improving their symptoms and creating structure in their lives. Our procedure was integrated as part of the treatment component ‘Healthy Lifestyle’. This component is focused on topics such as relaxation, managing finances, nutrition and the patients' social network. Normally, the treatment only contains one session about the patients' social network, but for our research 4 sessions of 90 min each were dedicated to the patients' social life. Our sessions were accompanied by two therapists who were responsible for a few different treatment components. Next to that, each of them was also a personal therapist for one of the patients outside of the group sessions.

#### 2.2.2 Case study 2 (CS2): transition group from inpatient treatment

The second case study took place in the transdiagnostic department. Specifically, we collaborated with the team of therapists responsible for the transition from inpatient treatment to daily life outside the clinic. The team included a total of five clinicians with varying functions. Patients in this transition group follow treatment on-site for one day a week to support their adjustment. In contrast to the group from the previous case study, this group was somewhat older, highly educated and followed regular employment at the time of the study. Part of our sessions (onboarding, network assessment) were accompanied by one therapist of the team, mainly to support data collection. Overall, therapists were less involved in CS2 than in CS1, as the full team only joined the evaluation.

#### 2.2.3 Case studies 3 & 4 (CS3 & CS4): individual treatment

CS3 and CS4 were with two young adults receiving individual outpatient treatment in the same department in which CS1 took place. Almost weekly, these two patients follow individual treatment sessions. The patient in CS3 participated next to her individual sessions also in regular group therapy sessions, but she took part with her individual psychotherapist. The patient in CS4 was only in individual therapy. The personal therapists of the patients were involved throughout the whole research procedure.

### 2.3 Procedure

If a patient (or group of patients) wanted to participate, researchers would contact the patient(s) to explain the study procedure in more detail. Subsequently, the researcher(s) joined a therapy session to sign the informed consent and discuss the complaints and treatment goals of the patient(s) to set up a partly personalized ESM diary. Directly after this session, a researcher assessed the social network of the patient in an interview (for details see section 2.4.1) and explained how to complete the ESM assessments. In the group trajectories each patient was interviewed by either a researcher or a therapist who guided the patient through the questions and recorded their responses. On the following day, a 14-day ESM period started, which assessed daily social interactions and complaints (for details see section 2.4.2). After that, a researcher joined a second therapy session to provide feedback to both patient and therapist using an interactive feedback report summarizing the PSN and ESM data (for details see section 2.4.3). Again, each patient in the group trajectory received individual attention from a therapist or researcher to explore their feedback report together.

**Fig. 1:**
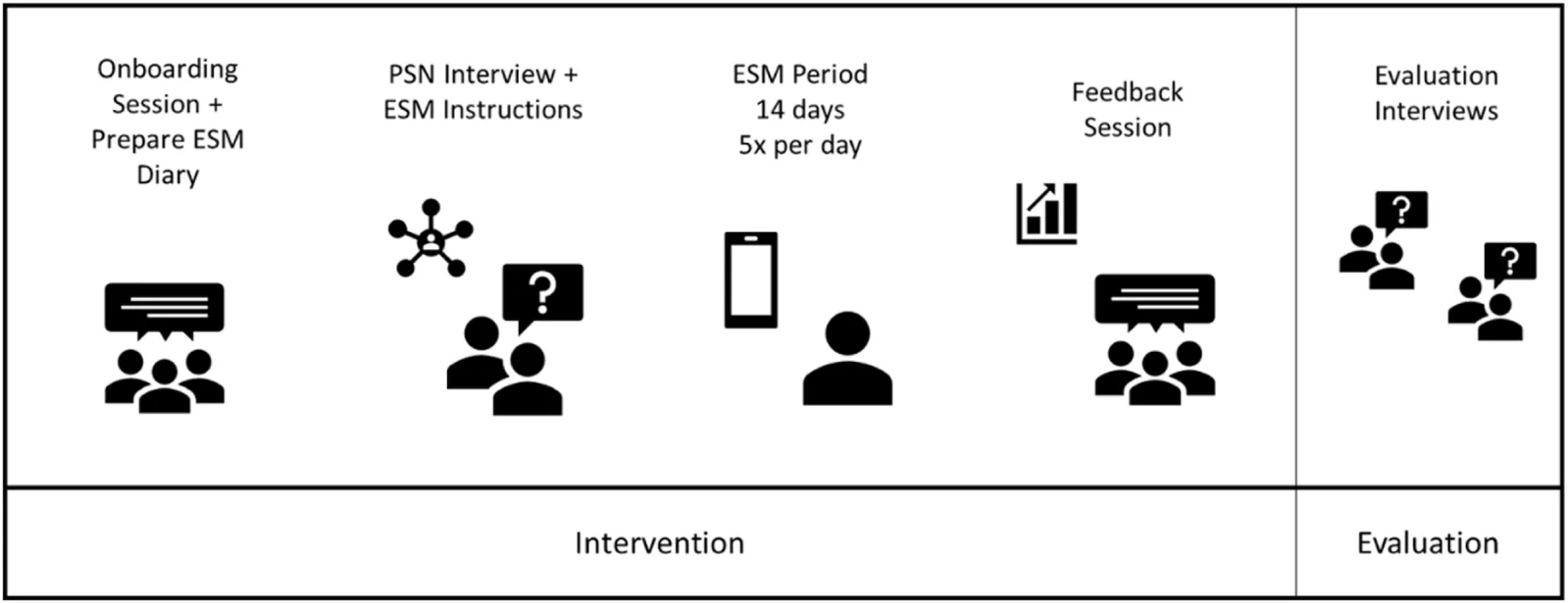
Procedure overview.

To evaluate the intervention procedure, we conducted semi-structured interviews with the therapists and patients separately (for details see section 2.4.4). An overview of the entire trajectory is depicted in Fig. 1. After every session, the two researchers doing data collection (MS and SW) discussed how the session went. These reflections were also written down to facilitate reflexivity and support analyses.

### 2.4 Materials

All materials and analysis code to generate the feedback report can be found on our Open Science Framework (OSF) repository (www.osf.io/jqdr9). Additionally, assessment materials are also provided in the supplementary materials.

#### 2.4.1 Personal network interview

PSN are assessed by either surveying or interviewing the participant about their social relationships (see McCarty et al., 2019) for an overview of PSN methodology). Due to the lack of approved network data collection software within the mental healthcare facility, we used an in-person interview supported by visualizations on a whiteboard. The first stage of a PSN data collection is name generation in which participants are prompted to list names of relevant network members. We used the following prompt: *“Name the most important people in your life. Think of people with whom you have regular contact and also people with whom you do not, but whom you could contact.“* Patients wrote each name on one magnet. The maximal number of network members was 25, a commonly recommended network size to capture not only close relationships but also weaker ties (Stadel and Stulp, 2022; McCarty et al., 2007; Golinelli et al., 2010). The second stage of a PSN data collection contains the so-called name interpreter questions which the patient answers about each network member: We asked a total of ten questions about characteristics of the network members, such as the patient's relationship with these people, and how often the patient has contact with them. A full list of the original Dutch questions and their English translation can be found in our supplementary materials. We printed each question and its answer scale on a small sheet which were both attached to the whiteboard while answering the question. Patients were asked to sort the magnets (i.e., network members) into the appropriate response category by moving them on the whiteboard. Such visual methods have been shown to improve data quality and participant experience during a network interview (Stark and Krosnick, 2017). The last step of a PSN assessment is to ask which network members know each other. We defined a connection as network members who know each other's names, faces and have regular contact with each other (at least once a year). During the PSN interview, the interviewer used a pre-formatted Excel file to record the participant's responses through all stages, so that the data could later be visualized in the patient's feedback report.

#### 2.4.2 ESM

In several mental healthcare institutions in the Netherlands, the ESM tool PETRA (**PE**rsonalized **T**reatment by **R**eal-time **A**ssessment (https://umcgresearch.org/petra/home;Bos et al., 2022) is available to therapists and researchers via the routine outcome monitoring software integrated into the electronic patient database. PETRA allows setting up an ESM diary for patients currently in treatment and sends an SMS to the patients’ mobile phone which contains a link to the assessment. Given its specific tailoring to the clinical setting, including secure data storage, we used PETRA for our ESM assessments.

The ESM period lasted 14 days and included 5 prompts per day at semi-random moments (i.e., 70 assessments in total), which took place during a 10-h time window (e.g., 9:30 until 19:30). The start time (and thereby also the end time) was determined by the participant according to their daily rhythm.

All ESM items that we used are part of the PETRA item databank (for details we refer to Bos and colleagues (Bos et al., 2022)) The ESM diary contained a set of items about mood and social interactions since the last assessment that was the same for all patients. Additionally, each patient could select three items that matched their treatment plan (e.g., their most important complaints). For this, participants received a list of available items (see the supplementary materials for a full list of all fixed and optional ESM items and their response scales).

#### 2.4.3 Feedback report

Each participant received a slightly modified and translated version of the feedback report presented by Stadel and colleagues (Stadel et al., 2023). Before the feedback session, the researcher prepared the digital interactive version of the report and a printed summary version with short explanatory texts that the patient could take home. The explanatory texts outline how to read the graphs rather than present the patients with conclusions about their symptoms or social life. The interpretation of the results was done in discussion together with the patient (and therapist) during the feedback session. The digital version of the report also contains several ways to interact with the graphs (e.g., selecting whether to only look at face-to-face social interactions or also include online ones, selecting different symptom or interaction quality ratings to be displayed on the Y-axis of the graphs, filtering out interactions with specific partners, and being able to click on specific social interaction moments to display details about that interaction). The printed version of the feedback report contains screenshots of a selection of visualizations from the digital report (an example of the printed feedback report is provided in the supplementary materials).

#### 2.4.4 Evaluation interviews

We evaluated our intervention in separate patient and therapist interviews conducted by MS. For CS1 and CS2, evaluation interviews were conducted in patient and therapist groups, resembling a focus group discussion. In these cases, SW supported the evaluation. During the patient evaluations, we first used a short questionnaire assessing demographic variables as well as clinical characteristics. Subsequently, we asked the patient to share their opinion about each part of the research and their overall impression of the utility of the intervention for their treatment. Therapists were asked to evaluate the use of the intervention for the current case, to reflect on the broader clinical utility of the intervention and describe what would be needed to implement the intervention in clinical practice. If the participants consented, the sessions were audio recorded. Otherwise, the researcher took detailed notes.

### 2.5 Analysis

We qualitatively evaluated study experience using codebook thematic analysis (Braun and Clarke, 2021), combining deductive coding based on predefined domains with inductive coding to identify unanticipated patterns across participants’ accounts. This approach was well suited to our case series, in which patients, therapists, and researchers each offered distinct perspectives on the feasibility, usefulness, and possible improvement of the intervention. We therefore triangulated these viewpoints in the analysis. Recordings of patient and therapist evaluations were transcribed using a local version of WhisperAI (OpenAI, 2022), checked and pseudonymized by SW, and analyzed together with researcher notes in Atlas.ti Web (Version 25.0.1) by MS and SW. After data familiarization, we defined six overarching domains to guide coding: motivation for using the intervention, effects of use, positive feedback, encountered problems, suggestions for improvement, and important considerations for implementation. Themes were then developed through line-by-line coding and subsequent collation of codes, while allowing additional codes outside these domains, where warranted by the data. During this process, we used elements of the Framework Method, a codebook-based approach to thematic analysis (Ritchie et al., 2013), including participant-by-session summaries and matrix displays, to compare domains within and across participants and cases, with particular attention to contrasts between patient and therapist evaluations within the same case. Alongside the qualitative results, we present and interpret compliance and data quantity for the PSN and ESM data. Quantitative data was handled in R (R Core Team, 2024).

## 3 Results

During our analysis we noticed that the six initial domains could be summarized in three, as the domains positive feedback, problems and suggestions overlapped largely in their content and all spoke to the larger domain of points to consider for the implementation. The results are structured as follows: We first describe the themes falling under the domain motivation – why did patients and therapists take part in the study and what did they hope to gain from our intervention? Second, we present compliance results. Third, we describe the effects our intervention has had (i.e., what it delivered for patients, therapists and their shared treatment process) based on their subjective evaluation and researcher observation, and lastly, we summarize additional topics to consider for implementation of the intervention.

### 3.1 Motivation

Patients and therapists described several motivations for participation. While one patient chose not to participate because she anticipated little new insight in social context, noting, *“I already have the insight myself, well, of which people I do get energy from and which not”* (001, CS1), others were curious to learn more about their social context and how it relates to their complaints. For four patients, their social context was central to their treatment. It is important to note that patients during the first case study tended to avoid challenging situations and were therefore nudged somewhat towards participation by their therapist. In the other case studies participants were intrinsically motivated to participate.

Therapists across case studies emphasized that social context is insufficiently explored in routine care. For example, one therapist from CS1 mentioned: *“I think that for us too it can still become a kind of forgotten [topic]. While we […] know how important it is [we] do not know exactly what that social network looks like. Look, you know superficially whether someone plays soccer or whether they have friends or no friends or see people online or not online. But what it looks like exactly is not clear.”*, while expressing strong confidence that the feedback from our intervention could be transformative: *“if you could feed that back to someone and could show, hey, at the moment that you seek support from another person, we simply see factually that you are doing better. That in theory could be mind changing*.*”*

Therapists from CS1 highlighted the relevance of social context, especially for young adults, whose networks are still developing, and therapists from CS2 emphasized a similar importance during the transition out of inpatient treatment, hoping our method would help patients make more conscious social choices. Therapists from CS2 further noted the importance of seeing the social context of some patients visible “*black on white*”. This idea of obtaining ‘hard’ data, potentially to challenge patients is also shared by the therapist from CS3 (*“it is also nice to see, can we show her a slightly more nuanced picture? Is what she thinks actually correct […] That you can especially sometimes show patients another perspective.”*) and CS4 (*“My hypothesis was that he labelled some contacts more positively than I thought they were”*).

### 3.2 Compliance

Seven out of nine patients completed the entire study procedure with varying degrees of compliance. As mentioned above, patient 001 opted out of the ESM period and therefore only received feedback on her social network. Patient 003 stopped the ESM period after two days and did not participate in the evaluation session either – the therapists later clarified that her symptoms were too severe to participate due to an acute crisis. An overview of compliance and the obtained data quantity in terms of ESM and PSN data is displayed in Table 3. A stark contrast is visible between CS1 and the remaining patients, matching the differences in motivation to participate and the fact that this group was at the beginning of their treatment with complaints still being relatively high. The group of CS2 showed higher compliance overall and larger social networks, despite minimal therapist involvement in their trajectories. Compliance was highest in the two individual case studies in which patient and therapist completed the whole trajectory in close collaboration (i.e., discussing the set-up of the diary as well as the feedback report together in a session).

**Table 3:** Compliance results.

Patient	Case Study	Network size (+ extra interaction partners)	ESM compliance	Number of reported social interactions
001	1	25	-	-
002		14	30%	14
003		22	4%	0
004		12 (+1)	31%	17
005	2	23 (+9)	77%	43
006		22 (+10)	70%	38
007		22 (+6)	43%	16
008	3	24 (+3)	93%	38
009	4	25 (+11)	90%	32

### 3.3 Intervention effects

Based on the evaluation interviews, the use of our intervention had three kinds of effects: patients feeling like the feedback confirmed their self-perception, patients and therapists gaining more insight into complaints and social context, and patients feeling confronted, which seemed to be a key element of the intervention. However, often these three appeared somewhat intertwined.

Many patients expressed they did not get new insights from the feedback, but that it rather confirmed their self-perception (*“Those are things that, in a way, you do already know.“* – 008 CS3). Despite that, the feedback was still perceived as useful for the therapist (e.g., *“Because they only see a small part of us, of course. And we do tell them some things. But imagine that you really would just fill in the diary. And truthfully. Then yes, I think it really could be supportive. For the treatment.”*- 001, CS1 or *“if you have people around you who are important, or you say a friend instead of the name. And now you really have to link a name. You don't do that so quickly in a treatment.”* – 009, CS4). In some cases, patients seemed to have gained insight through the assessments rather than the feedback, as these prompted them to be more mindful (*“It also asks you to pause, so in that sense it also teaches you to do things alongside your treatment, to pause. It helps with that too, I do see that.”-* 004, CS1).

Often, when patients named a confirmatory effect, they also noted the difference between *“knowing something deep down”* and being fully aware and ready to bring it into therapy. Several patients emphasized that it made a difference “*seeing it”* in our feedback report rather than just *“somehow”* knowing it (e.g., *“I actually never talk with my friends about difficult things or so. I did know that, of course. But then, yes, when you see that …”-* 002 CS2). Our intervention seemed to have achieved present awareness and the feedback acted both as a visualization and a communication device between patient and therapist (*“normally we talk about my family, but I'm not around them that much … And I do know that … But yes, now [my therapist] could see it as well for once*” – 008, CS3).

In CS3, we observed an interesting paradox related to insight: Patient 008 claimed that she did not gain new insights from the study, but then later in the interview proceeded to describe concrete new insights she gained: *“It did affect me for a moment, and I also found myself thinking a lot about some friends, like, oh, do I actually recharge with them? I always say I do, but with some things you just started to pause a bit more.”* Thus, patients may not always recognize new insight as such.

In this case, the intervention also delivered useful insights for the therapist. She particularly noticed the more procedural descriptions of the patients’ social interactions (*“There was little color to her [interaction descriptions]. I think she also finds that one of the most difficult things—giving her own color to the contact, to herself […] you can then take that with you for the therapy goals.“*) and described several other points she noticed in the feedback to be discussed with the patient in future sessions. Thus, in their case the intervention delivered useful input for therapy for both the patient and the therapist.

Another patient 004 and her therapist from CS1 similarly benefited from our intervention. This patient gained several important insights (e.g., *“Friend A just drains so much out of me. While friend B didn't do that at all. And from that I was able to gather that, for example, I'm going to tell friend A more often: I don't want this anymore for now, I'll leave it at this, I'll keep it short.”*). In this case, the intervention even prompted behavioral change. She did not only change her interaction with friends, but also took the feedback report to her family, which helped her communicate her struggles and fostered connection. Her therapist noted: *“I'm getting a lot of insight into who is in [the patient's] network, but also into the way they are in her network. And that a very large part of it is online, and that a whole lot of them actually only cost energy, don't give any energy. So yes, I found it very clarifying to map it out like that.”*

For the intervention to be effective, patients, however, needed to engage in what they perceived as confrontation (*“You had to place people into categories; it was somewhat painful, and you could even be ashamed of it.”* – 005, CS2). The need for engaging in confrontation is central to psychotherapy according to the therapist from CS3: *“Yeah, then it might be quite painful to look back at, and at the same time I think, yes, that's exactly what you learn from, I think. Therapy is also always a bit unpleasant, toward the pain.”.* For some patients, however, engaging in the diary assessments was seen as too confronting, particularly in CS1: Patient 001 decided to not participate at all and 002 avoided completing the ESM assessments when he had challenging social interactions or generally a bad day (e.g., *“But I did notice, in those two weeks I had a really difficult weekend. Yeah. And then I noticed, yeah, I really don't feel like dealing with that.”*). During the evaluation interview, 002 regretted his lower compliance and recognized the importance of confronting oneself: *“it might actually be very valuable if you fill it in when you're feeling bad. Though yes, it may be harder, but I think the results are even more valuable if you know them at that moment.”*. A key element for the intervention to be effective, thus seems to be achieving the right level of confrontation for a particular patient.

### 3.4 Implementation

With regards to the further implementation of our intervention in clinical practice, the evaluation interviews revealed several important points: All therapists agreed that it is most useful in early phases of treatment, after they had a chance to build sufficient rapport with a patient to engage in the necessary confrontation this intervention comes with. Both patients and therapists saw repeated use of the intervention later in treatment as valuable as well.

When using the intervention, patients cannot be in an acute crisis while completing the intervention, as was the case for patient 003. A particular challenge seemed to be patients struggling with avoidance as in CS1. Such patients need continued motivation by therapists to engage sufficiently with the assessments. Our intervention appeared most successful in individual applications in which therapists and patients set up the diary and discuss the feedback collaboratively, which seemed to help patients to dare to be confronted.

We, however, observed that patients were not always driven to bring in outcomes from the feedback report in other group sessions (*“I haven't literally heard it back anywhere. It has stayed out of the therapies”* – therapist CS2) – therefore, follow-up actions of a therapist engaging with the feedback report are needed for optimal use. This may be supported by adding the feedback report to patient dossiers and making the digital report accessible by therapists without researcher involvement.

According to the therapists from CS1 and CS3, the time investment for the intervention part was feasible given that it took only two therapy sessions: One session to discuss the set-up of the diary and one session for the feedback report. Thus, when given software and support staff to carry out the social network assessment, an automatically generated feedback report and training, therapists consider the use of our intervention feasible and useful for daily clinical practice. Several therapists mentioned that they were motivated to use the intervention with other patients of theirs.

Within the transition from in-patient treatment (CS2), therapists are generally less involved and therefore would prefer our intervention to be standalone for patients. This is contrary to one patient's wish to at least discuss the feedback report with a therapist (*“I do feel the need to talk with [a therapist] about the results”* – 005, CS2). In theory, a patient could take discussion topics from our intervention to other therapy sessions. However, according to the therapists, this patient has not brought up anything following the feedback session. Thus, also in this setting, it would be desirable to have therapists involved in the feedback session or that therapists follow up on outcomes during other sessions.

## 4 Discussion

In a series of four mixed-methods case studies, we explored the feasibility of a novel intervention capturing the social context of psychotherapy patients. The intervention consisted of two components (1) the assessment of social context using the experience sampling methodology (ESM) and personal social network data collection (PSN) and (2) a feedback report summarizing the patients' social context. We explored feasibility and utility of this intervention in group and individual psychotherapy of young adults with severe mental health issues who were either in partial in-patient treatment or transitioned out of full in-patient treatment. Evaluation was carried out using qualitative data from semi-structured interviews with patients and therapists as well as researchers' reflexive notes taken while delivering the intervention, and quantitative data regarding patients’ compliance with the assessments that were part of the intervention.

Based on preliminary evidence by Stadel and colleagues (Stadel et al., 2023) we expected the intervention to be most promising in settings where the feedback is discussed between the patient and therapist. In line with this expectation, useful engagement with the intervention appeared most feasible in individual therapy, when the therapist and patient collaboratively set up the diary and discussed the feedback. This matches results from previous research highlighting that ESM-based feedback can facilitate communication between patient and therapist (Bos et al., 2020) and improve the patient-therapist relationship (Bos et al., 2019). While our results indicate that also group applications may be feasible, they require similar therapist engagement and need to be carefully integrated into the treatment process, as Bartels and colleagues (Bartels et al., 2023) recommend ESM-based feedback to always be discussed face-to-face.

Our results show that the intervention may also lead to new insights in patients, whereby insights can range from more subtle confirmatory effects to recognizing important behavioral patterns, such as which kinds of social interactions are beneficial for them. The way participants described the effects of the intervention points at them making a shift from previous intellectual insights towards more emotional insights which lay the foundation for behavioral change (Ellis, 1963). In line with other ESM research (Stadel et al., 2023; Bos et al., 2020; Bakker and Rickard, 2018), this process seemed to be facilitated by the self-reflection prompted by the assessments or through discussion between patient and therapist, rather than viewing the feedback report without further therapeutic action.

Interesting about our results is that also in some cases in which the patient did not show high compliance with the diary assessment, useful insights could be gained, and patients could be motivated to make more informed choices about social behavior in daily life. Rather than the completion of many assessments, one of the key mechanisms of the intervention appears to be that patients are confronted with potentially uncomfortable situations. That working mechanism is connected to the main principles and techniques in CBT specifically in exposure therapy (Beck, 1979) and more general mechanisms of psychotherapy (Alberti, 2018). Thus, for the intervention to be useful, patients need to be able to be confronted by their therapist, who uses the feedback report as a tool to confront the patient. Given its similarity to exposure therapy in working mechanisms, a strong therapeutic alliance between patient and therapist is important for our intervention to be successfully applied (Buchholz and Abramowitz, 2020; Podolan and Gelo, 2023; Moeseneder et al., 2019).

Beyond the possibility to use the feedback report for confrontation, our intervention delivered the therapists with detailed knowledge about the patients’ daily social life. Considering the importance of social context for psychological functioning (Southwick et al., 2016) such knowledge may aid the further treatment process. The feedback report can be used to set additional treatment and recovery goals, more specifically on perceiving and seeking social support (Scardera et al., 2020; Nicaise et al., 2022). Thus, even though it might not be effective to use our feedback as a standalone intervention given to patients without further therapeutic action, the potential uses for further treatment with the therapist are manifold.

### 4.1 Limitations and future research

Our results must be interpreted in the context of some limitations. First, during this pilot, the intervention was heavily supported by the researchers. Future applications without researchers need an automatically generated feedback report which is integrated into the digital infrastructure of the mental healthcare facility. Next to that, therapists need to be trained in the use of the intervention and how to follow-up on its outcomes. Part of this training needs to include how to safeguard themselves and patients from overinterpreting the report. During our analysis we noticed several references to the report as ground truth (“*black on white*”). While many patients and therapists were nuanced in how they viewed the data (e.g., emphasizing that it only refers to two weeks of their lives and that they did not meet everyone in their network), some participants seemed to have the assumption that our intervention delivers ‘hard data’ that can be interpreted as unquestionable facts. We would like to emphasize that the presented data is self-reported by the patient and covers only a limited period. There can be discrepancies in how the therapist or researcher interprets questionnaires and how the patient was using it (Schorrlepp et al., 2025; Truijens et al., 2022, 2023). Therefore, appropriate training to critically appraise the data represented in a feedback report and a shared discussion between patient and therapist to calibrate interpretation are essential.

Lastly, it needs to be noted that we conducted only a small-scale exploratory study within one mental healthcare institution. We encourage the reader to critically reflect on the clinical context in which they would like to use the presented intervention and whether our findings could be transferred. While our study was able to give an indication regarding the utility and working mechanism of the intervention (i.e., the why and the how), larger scale research to quantitatively evaluate feasibility, acceptability and intervention effects (e.g., what impact does the intervention have on symptoms and functioning outcomes in the short- and long-term) are needed.

## 5 Conclusions

This study explored the feasibility and utility of a novel intervention for capturing patients’ social context by combining experience sampling methodology with personal social network data collection in clinical practice. Across four case studies spanning both group and individual psychotherapy, we found that the intervention appeared most feasible and useful in individual therapy, although useful integration is achievable in group setting, given sufficient therapist involvement. Both patients and therapists identified meaningful benefits arising from the assessment process as well as from the individualized feedback reports. Successful implementation, however, depends on active therapist involvement, dedicating at least two therapy sessions to the procedure. Finally, further automation of procedural steps and structured therapist training will be essential to support future quantitative evaluative research and implementation of the intervention.

## Declaration of generative AI and AI-assisted technologies in the manuscript preparation process

During the preparation of this work the authors used ChatGPT 5.1 to refine writing for selected paragraphs and the abstract. During revisions ChatGPT 5.4 was used for the same purpose. After using these tools, the authors reviewed and edited the content as needed and take full responsibility for the content of the published article.

## Declaration of competing interest

The authors declare that they have no known competing financial interests or personal relationships that could have appeared to influence the work reported in this paper.
