# Supplementary material for: Assessing psychotherapy patients’ social context through personal networks and experience sampling: A mixed methods case series exploring feasibility
Source: Neurosci Appl. 2026 Sep 9;5:107029. doi: 10.1016/j.nsa.2026.107029 (PMC13589085; doi:10.1016/j.nsa.2026.107029)
Supplement: Supplementary file 1 [file mmc1.pdf]

Supplementary Materials: ***Assessing Psychotherapy Patients' Social Context Through Personal Networks and Experience Sampling: A Mixed Methods Case Series Exploring Feasibility***

S.1 Personal Social Network Questionnaire

Assessment Stage	Original Item (Dutch)	Translated Item (English)	Response scale	Instructions / Assessment Form
Generating names of network members	Benoem de belangrijkste mensen in je leven. Denk aan mensen met wie je regelmatig contact hebt en ook aan mensen waarmee je dat niet hebt, maar met wie je wel contact zou kunnen opnemen.	Name the most important people in your life. Think about people with whom you have regular contact, and also people with whom you don't have that, but whom you could contact.	*Typed name*	*The participant types names into the Excel sheet while the researcher writes each name on a separate magnet.*
Characteristics of network members	Wat is je relatie met deze persoon?	What is your relationship with this person?	*Kind (Child)*<br>*Partner (Partner)*<br>*Ouder (Parent)*<br>*Broer of zus (Sibling)*<br>*Ander familielid (Other family member)*<br>*Vriend (Friend)*<br>*Collega (Colleague)*<br>*Kennis (Acquaintance)*<br>*Begeleider/behandelaar (Advisor / therapist)*	*Participants select the appropriate category in the Excel sheet using a drop-down menu. In case someone belonged to multiple categories, participants were asked to select the one that fit their relationship with this person best.*
	Hoe lang kennen jullie elkaar al?	For how long have you known each other already?	*0 = Minder dan 1 jaar (Less than one year)*<br>*1 = Tussen 1 en 5 jaar (Between 1 and 5 years)*<br>*2 = Meer dan 5 jaar (Longer than 5 years)*	*The whiteboard is divided into three columns by drawing two vertical lines. Then participants sort all the magnets in the appropriate column.The researchers record the applicable responses in the Excel sheet.*

	Hoe vaak heb je **face to face** contact met deze persoon?	How often do you have face to face contact with this person?	*0 = < 1 keer per maand (> once per month)*<br>*1 = +/- 1 keer per maand (+/- once per month)*<br>*2 = Paar keer per maand (A few of times per month)*<br>*3 = Paar keer per week (a few times per week)*<br>*4 = Dagelijks (Daily)*	*The whiteboard is divided into five columns by drawing four vertical lines. Then participants sort all the magnets in the appropriate column. The researchers record the applicable responses in the Excel sheet.*
	Hoe vaak heb je via **digitale apparaten** contact met deze persoon?	How often do you have contact via digital devices with this person?		
	Ik bespreek persoonlijke zaken met deze persoon.	I discuss personal topics with this person.		
	Deze persoon ondersteunt me wanneer ik emotionele steun nodig heb (bijv. troost, sympathie en aanmoediging).	This person supports me when I need emotional support (e.g., comfort, sympathy and encouragement).		
	Ik kan mezelf zijn bij deze persoon.	I can be myself with this person.		
	Tijd doorbrengen met deze persoon **kost** me energie.	Spending time with this person costs me energy.		
	Tijd doorbrengen met deze persoon **geeft** me energie.	Spending time with this person gives me energy.		
	Deze persoon geeft mij praktische of materiële ondersteuning.	This person provides me with practical or material support	*0 = Nee*<br>*1 = Ja*	*Researchers walk through the list of names and record the participant's yes/no response immediately in the Excel sheet.*

Ties between network members	Wie kent wie met naam en gezicht + wie heeft meer of minder regelmatig contact, tenminste 1x per jaar?	Who knows whom by name and face and has more or less regular contact - at least once per year?	*0 = no connection*<br>*1 = connection*	*Participants fill in 0 or 1 in each cell of the adjacency matrix in the Excel sheet.*

S.2 Fixed items in the ESM Questionnaire in PETRA (All assessed 5x per day semi-randomly)

PETRA Dimension	Original Item (Dutch)<br>Translated Item (English)	Original Item (Dutch)<br>Translated Item (English)	Scale	Extra Information
Stemming (mood)	Op dit moment voel ik me opgewekt	Right now I feel awake	*Visual Analogue Scale (1-100)*<br>Helemaal niet - Heel erg<br>Not at all - Very much	*These items are standardly in every PETRA diary and cannot be de-selected.*
	Op dit moment voel ik me somber	Right now I feel sad		
	Op dit moment voel ik me tevreden	Right now I feel content		
	Op dit moment voel ik me geïrriteerd	Right now I feel irritated		
	Op dit moment voel ik me energiek	Right now I feel energetic		
	Op dit moment voel ik me onrustig	Right now I feel restless		
Gesprekken	Heb je sinds het vorige meetmoment gesprekken gehad?	Did you have conversations since the last assessment?	Ja / Nee<br>Yes / No	*A conversation was defined as a social interaction with back and forth that took at least 5 minutes.*
	Sinds het vorige meetmoment, hoeveel gesprekken heb je gehad?	How many conversations did you have since the last assessment?	*Fill in a number*	
	Denk aan het voor jou meest belangrijke gesprek sinds het vorige meetmoment.<br>Met wie heb je dit gesprek gehad?	Think about your most important conversation since the last assessment. With whom did you have this conversation?	*Fill in a name*	*Participants were instructed to always use the same spelling for the same person (also across the network and ESM assessment).*

	Wat voor gesprek was dit?	What type of conversation was this?	face to face / digitaal<br>Face to face / digital	
	Hoe belangrijk was dit gesprek voor je?	How important was this conversation?	Visual Analogue Scale (1-100)<br>Helemaal niet - Heel erg<br>Not at all - Very much	
	Wat maakte dit gesprek belangrijk en waar ging het over?	What made this conversation important and what was it about?	*Type text*	
	Hoe lang duurde het gesprek?	How long did the conversation take?	*Type text*	
	Hoe plezierig was de inhoud van het gesprek?	How enjoyable was the content of the conversation?	Visual Analogue Scale (1-100)<br>Helemaal niet - Heel erg<br>Not at all - Very much	
	Hoe plezierig heb je het gesprek zelf ervaren?	How enjoyable did you experience the conversation itself?		
	In hoeverre beïnvloedde het gesprek je stemming?	To what degree did the conversation influence your mood?		
	In hoeverre beïnvloedde het gesprek je energieniveau?	To what degree did the conversation influence your energy level?		
	In hoeverre kon je jezelf zijn in dit gesprek?	To what degree could you be yourself during the conversation?		
	Ik voelde me gesteund door mijn gesprekspartner(s).	I felt supported by my interaction partner.		

S.3 Optional items in the ESM Questionnaire in PETRA (Patients could pick up to 3)

Domein	Vraag (Antwoordschaal is altijd: Helemaal niet – Heel erg)	Question (Response scale is always: Not at all - Very much)
Agitatie en stress	Op dit moment voel ik me gestrest	Right now I feel stressed
	Op dit moment voel ik me wantrouwig	Right now I feel suspicious
	Op dit moment voel ik me gespannen	Right now I feel tense
	Op dit moment voel ik me gejaagd	Right now I feel agitated
	Op dit moment voel ik me prikkelbaar	Right now I feel irritable
	Op dit moment voel ik me overprikkeld	Right now I feel overstimulated
	Op dit moment is het chaos in mijn hoofd	Right now there is chaos in my head
	Op dit moment ben ik snel afgeleid	Right now I am quickly distracted
	Op dit moment kan ik me goed concentreren	Right now I can focus well
	Op dit moment zie ik op tegen de rest van de dag	Right now I am looking forward to the rest of the day
Angst	Op dit moment voel ik me angstig	Right now I feel anxious
	Op dit moment voel ik me veilig	Right now I feel safe
	Op dit moment ben ik bang om alleen te zijn	Right now I am afraid to be alone
	Op dit moment ben ik bang dat ik een paniekaanval krijg	RIght now I am afraid to have a panic attack
	Op dit moment voel ik me onwerkelijk	Right now I feel unreal
	Op dit moment ben ik bang om de controle te verliezen	Right now I am afraid to lose control
Verminderde interesse	Ik heb zin in de rest van de dag	I am looking forward to the rest of the day.
	Op dit moment heb ik zin om met anderen te zijn	Right now I feel like being with others
	Op dit moment voel ik me lusteloos	Right now I am listless
(Hypo)manie	Op dit moment voel ik me bijzonder goed	Right now I am feeling especially good
	Op dit moment gaan mijn gedachten snel	Right now my thoughts are racing
	Op dit moment zit ik vol goede ideeën	Right now I am full of good ideas
	Op dit moment heb ik zin om met anderen af te spreken	Right now I feel like meeting other people
	Op dit moment kan ik snel schakelen	Right now I am quick on my feet
Piekeren	Op dit moment ben ik aan het piekeren	Right now I am worrying
	Op dit moment laten mijn gedachten mij niet los	Right now my thoughts are not letting me go
Positiviteit	Op dit moment voel ik me goed over mezelf	Right now I feel good about myself

	Op dit moment heb ik zin om met anderen te zijn	Right now I feel like being with others
	Op dit moment voel ik me ontspannen	Right now I feel relaxed
	Op dit moment voel ik me gewaardeerd	Right now I feel valued
Zelfbeeld	Op dit moment heb ik het gevoel te kort te schieten	Right now I feel like I'm falling short
	Op dit moment voel ik me nuttig	Right now I feel useful

S.4 Example of a Printed Feedback Report (Original Dutch Report)

Breng uw sociale leven in kaart!

Onderzoek naar het nut van de sociale context voor psychotherapie

Je relaties en sociaal netwerk

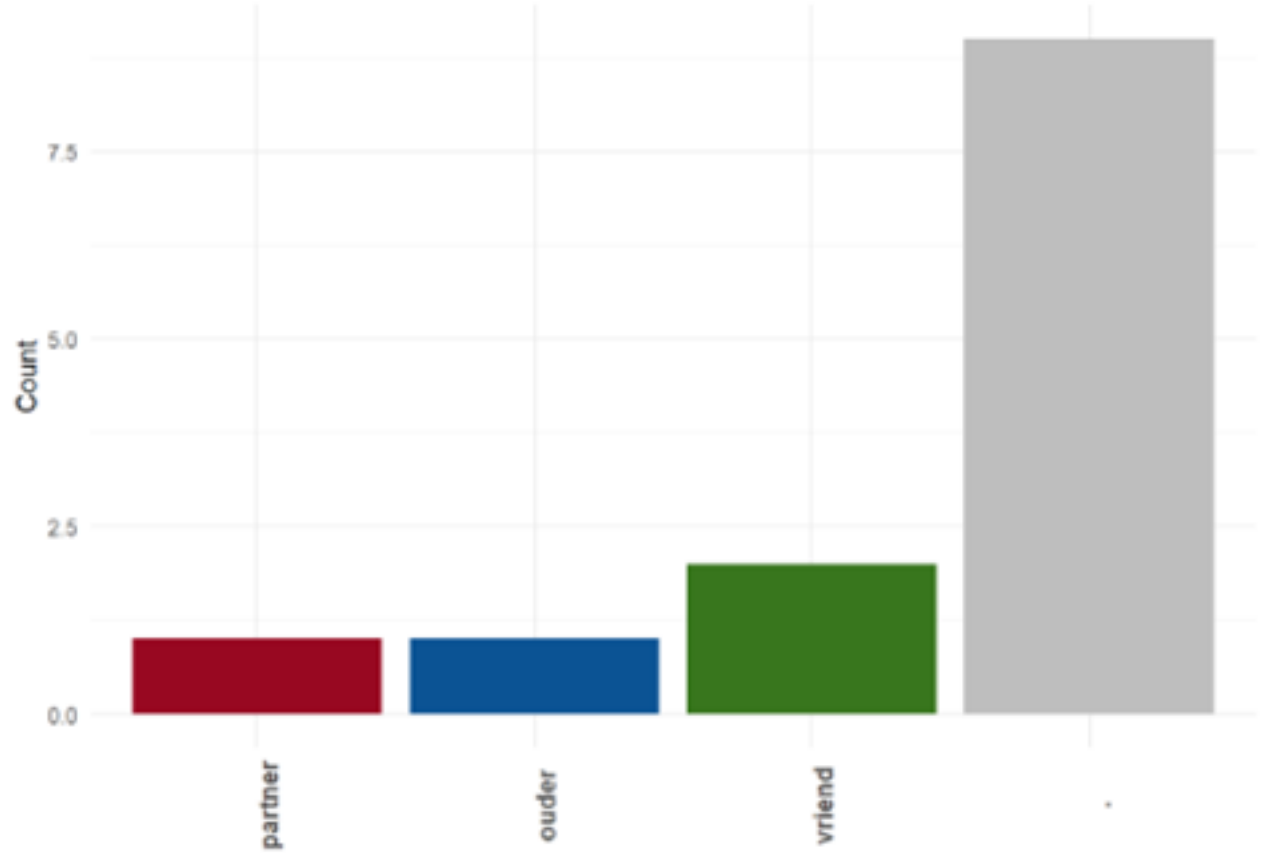

Uitleg: In deze grafiek zie je wat voor soort mensen je in je netwerk hebt (bijvoorbeeld hoeveel familieleden).

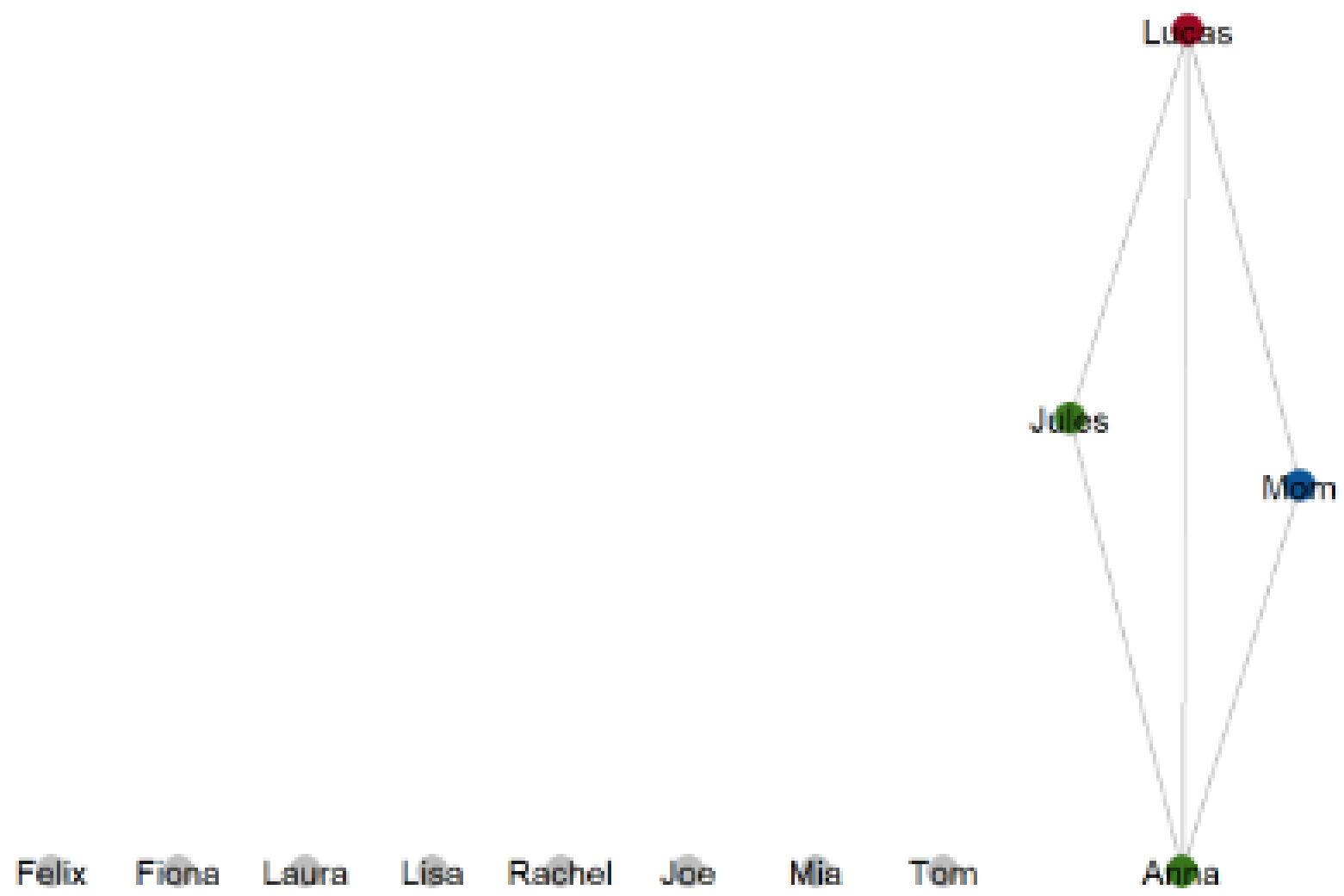

Uitleg: Hier zie je hoe de mensen in het netwerk met elkaar verbonden zijn (m.a.w. wie kent wie). De kleuren geven aan welke sociale rol een persoon heeft (bijv. vriend of ouder). Als er een persoon wordt weergegeven in een grijze kleur buiten het netwerk (linksonder), betekent dit dat je een sociale interactie had met deze persoon, maar dat je de persoon niet hebt opgenomen tijdens het meten van het sociale netwerk.

Naam	Relatie	Duur Relatie	Contactfrequentie face-to-face	Contactfrequentie digitaal	Emotionele steun	Jezelf zijn	Energie krijgen	Energie kosten	Praktische steun	Persoonlijke onderwerpen bespreken
Lucas	partner	Tussen 1 en 5 jaar	Dagelijks	Dagelijks	Bijna altijd	Bijna altijd	Vaak	Zelden	Ja	Bijna altijd
Anna	vriend	Meer dan 5 jaar	Paar keer per maand	Paar keer per maand	Vaak	Vaak	Vaak	Vaak	Ja	Bijna altijd
Jules	vriend	Meer dan 5 jaar	Paar keer per maand	Paar keer per maand	Vaak	Bijna altijd	Bijna altijd	Zelden	Ja	Bijna altijd
Mom	ouder	Meer dan 5 jaar	+/- 1 keer per maand	Paar keer per week	Bijna altijd	Bijna altijd	Bijna altijd	Zelden	Ja	Bijna altijd

Uitleg: Hier zie je een overzicht van elk netwerklid en wat je relatie met hen is. Over het algemeen wijzen meer groene vakjes op een positieve relatie. Dit zijn mensen die je steun (kunnen) bieden. Voor de mensen met meer gele of oranje vakjes kan het goed zijn om er bij stil te staan en na te denken waarom de relatie soms misschien wat minder positief is en of er mogelijkheden voor veranderingen zijn.

Kwantiteit en kwaliteit van je gesprekken

Gesprekken per partner

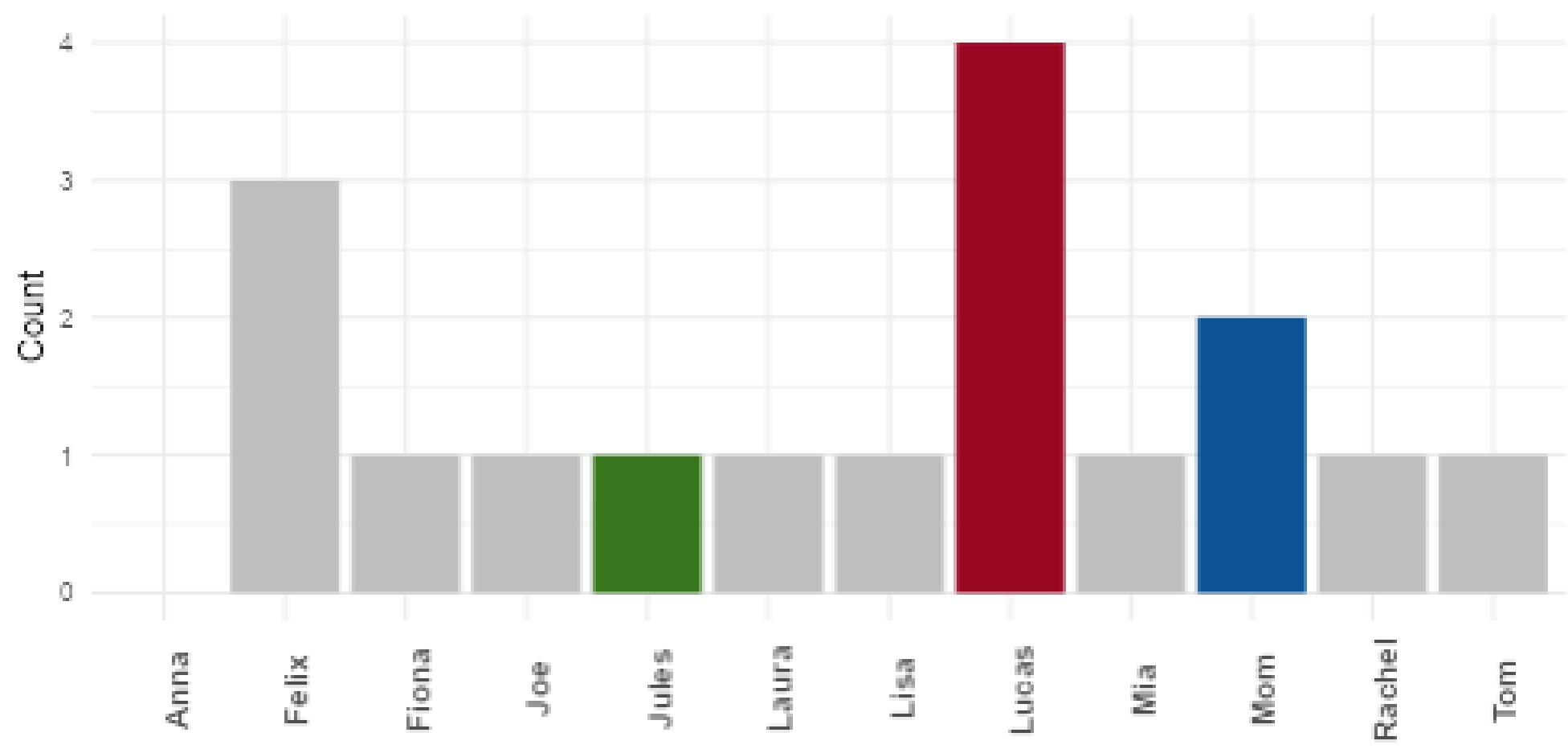

Uitleg: Hier zie je <u>hoeveel gesprekken je met wie had</u> tijdens de dagboekperiode. Houd er rekening mee dat dit slechts 14 dagen waren, dus dit geeft natuurlijk niet je hele sociale leven weer, maar het kan wel een indicatie geven.

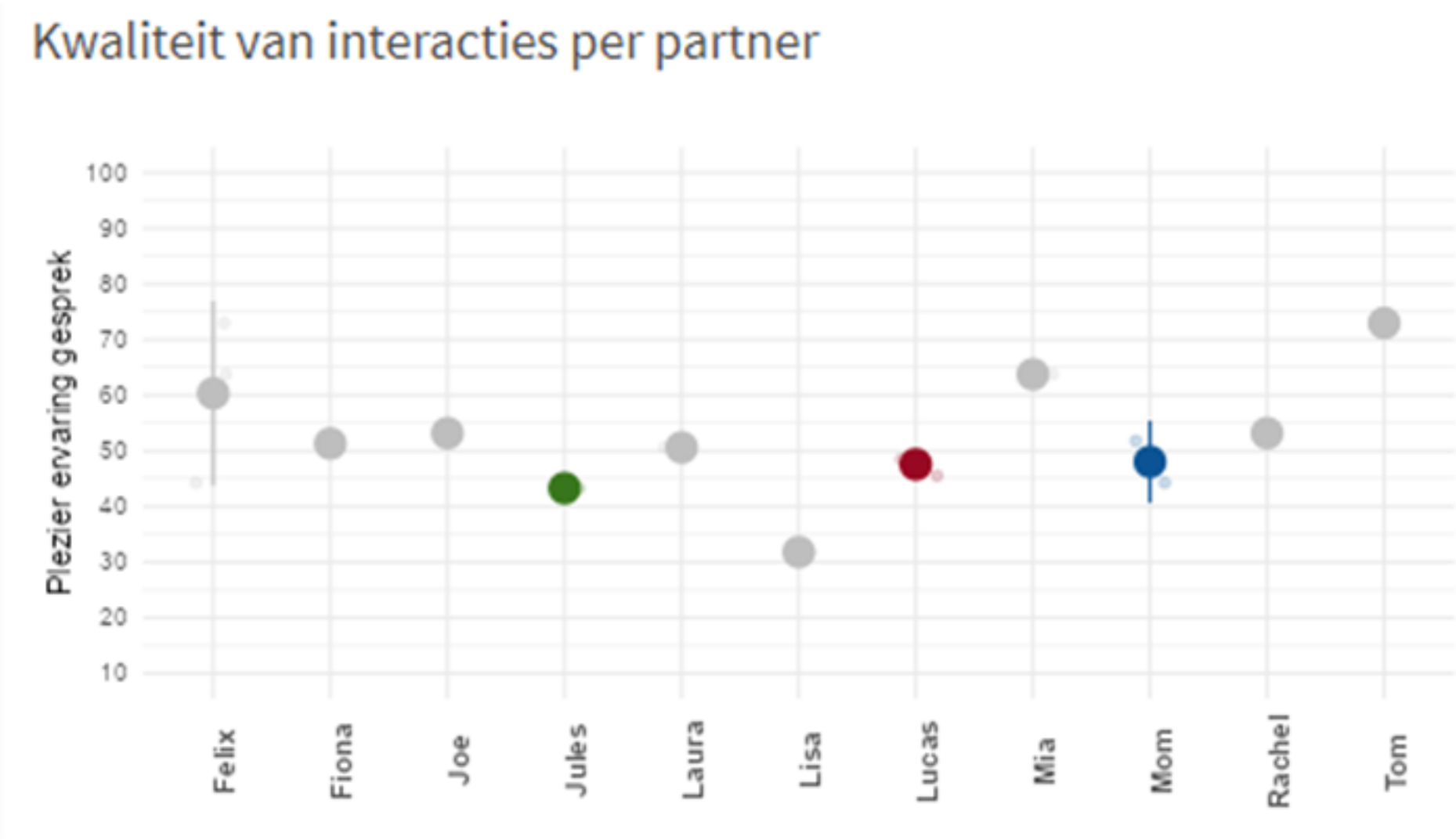

Uitleg: Hier zie je hoe je de gesprekken met elke persoon hebt ervaren. De grote stippen zijn een samenvatting van alle gesprekken met één persoon, de kleinere stippen eromheen geven elk afzonderlijk gesprek weer. Het kan zijn dat je met sommige mensen maar één gesprek had tijdens de 14 dagen, dan geeft de 'samenvatting' alleen die ene keer weer. Hoe hoger een stip staat, hoe positiever je de interactie hebt ervaren.

Gesprekken waarin je je gesteund voelde:

2	22-10-2024 12:35	Felix,Tom	Face-to-face	90	Follow up meeting about the work project with another colleague - the project is almost done	58.42
11	26-10-2024 09:22	Mom	Telefoongesprek	30	She advised me on how to handle a difficult situation at work	59.21

Gesprekken over moeilijke onderwerpen:

8	24-10-2024 17:47	Lucas	Telefoongesprek	10	I did not feel so well and wanted to talk about that with him. He was there for me.	44.76
12	27-10-2024 11:25	Lucas	Face-to-face	60	We had a big disagreement about our upcoming holiday plans	51.49

Uitleg: Dit zijn specifieke gesprekken die opvielen tijdens je dagboekperiode. Misschien zijn sommige hiervan nuttig om met je therapeut te bespreken. Je zou kunnen praten over de onderwerpen van het gesprek of ook over hoe je je wel of niet gesteund voelde.

Gesprekken en stemming / klachten

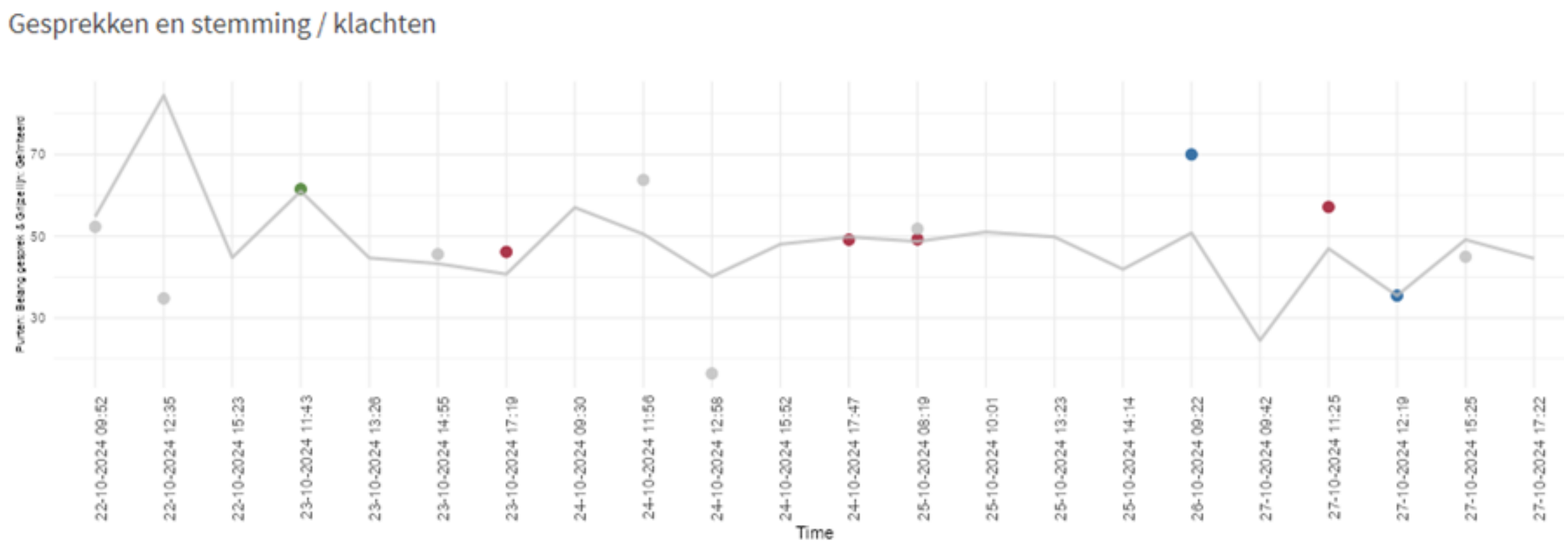

Uitleg: Deze grafiek laat zien <u>hoe geïrriteerd je je voelde</u> tijdens de 14 dagen van het dagboek (grijze lijn) en hoe gesteund je je voelde tijdens je gesprekken (stippen). Elke stip is één gesprek. Hoe hoger de stip, hoe meer je je gesteund voelde. Hoe hoger de lijn, hoe angstiger je je voelde. Let op de data onderaan de grafiek. Het kan zijn dat je dagboek metingen hebt gemist en dat deze grafiek daarom geen volledig beeld geeft.

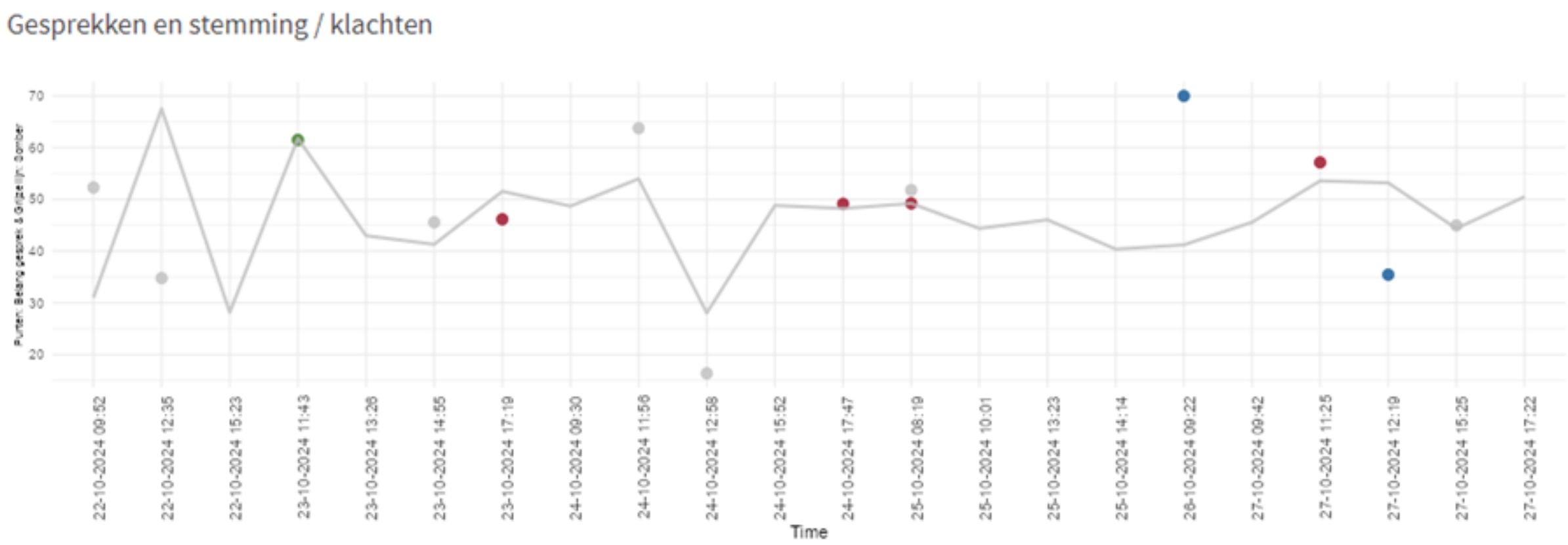

Uitleg: Hier is dezelfde grafiek, maar met een andere dagboek vraag als de grijze lijn: <u>'Op dit moment voel ik me somber'.</u>

S.5 Example of a Printed Feedback Report (Translated Explanatory Texts)

Breng uw sociale leven in kaart!

Onderzoek naar het nut van de sociale context voor psychotherapie

Je relaties en sociaal netwerk

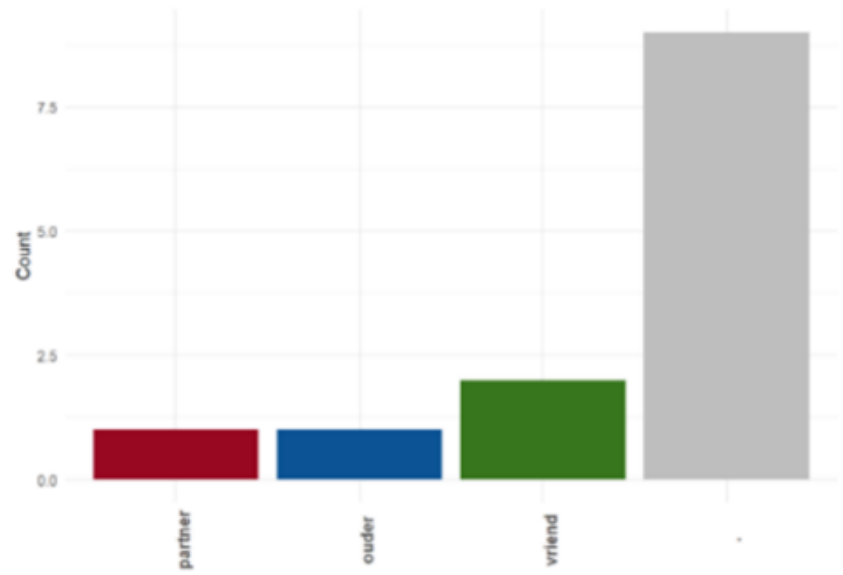

Explanation: In this graph you can see what kinds of people are in your network (for example, how many family members).

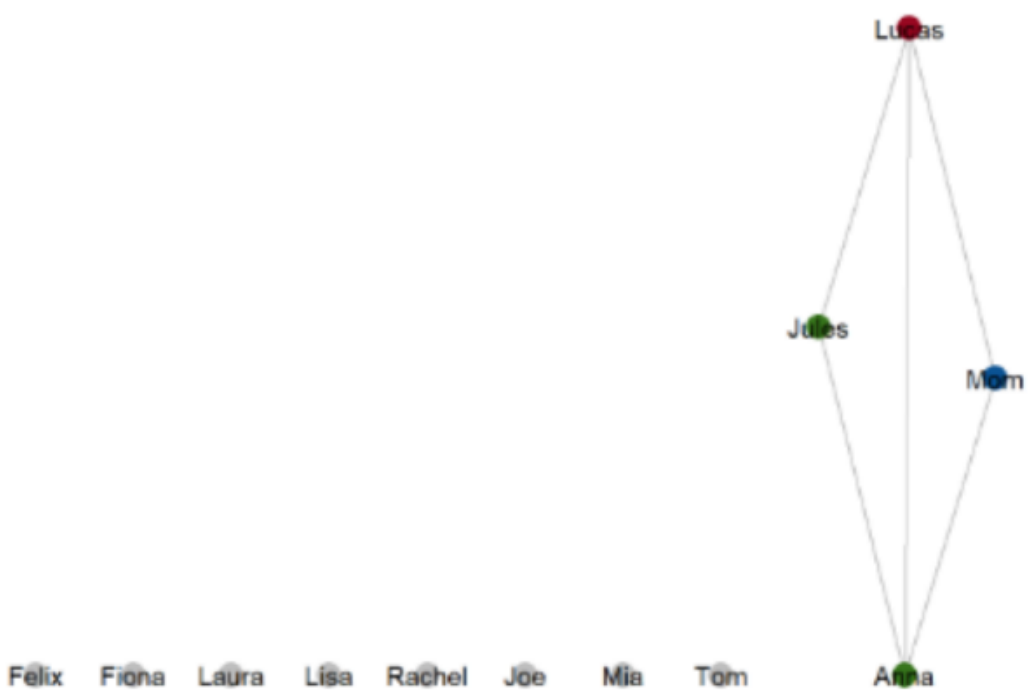

Explanation: Here you can see how the people in the network are connected to each other (in other words, who knows whom). The colours indicate the social role of each person (for example, friend or parent). If a person is shown in a grey colour outside the network (at the bottom left), it means that you had a social interaction with this person, but you did not include them when mapping your social network.

Naam	Relatie	Duur Relatie	Contactfrequentie face-to-face	Contactfrequentie digitaal	Emotionele steun	Jezelf zijn	Energie krijgen	Energie kosten	Praktische steun	Persoonlijke onderwerpen bespreken
Lucas	partner	Tussen 1 en 5 jaar	Dagelijks	Dagelijks	Bijna altijd	Bijna altijd	Vaak	Zelden	Ja	Bijna altijd
Anna	vriend	Meer dan 5 jaar	Paar keer per maand	Paar keer per maand	Vaak	Vaak	Vaak	Vaak	Ja	Bijna altijd
Jules	vriend	Meer dan 5 jaar	Paar keer per maand	Paar keer per maand	Vaak	Bijna altijd	Bijna altijd	Zelden	Ja	Bijna altijd
Mom	ouder	Meer dan 5 jaar	+/- 1 keer per maand	Paar keer per week	Bijna altijd	Bijna altijd	Bijna altijd	Zelden	Ja	Bijna altijd

Explanation: Here you can see an overview of each network member and what your relationship with them is like. In general, more green boxes indicate a positive relationship. These are people who (can) provide support. For people with more yellow or orange boxes, it may be helpful to pause and think about why the relationship might be a bit less positive and whether there are possibilities for change.

Kwantiteit en kwaliteit van je gesprekken

Gesprekken per partner

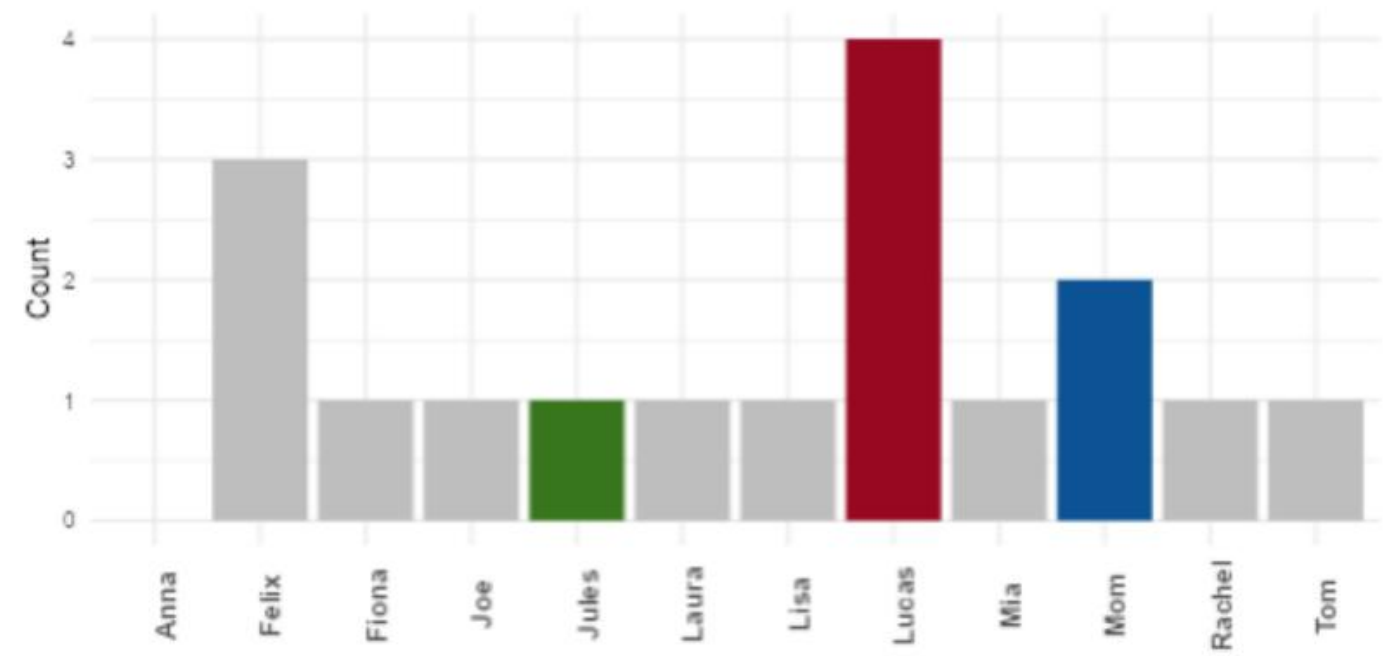

Explanation: Here you can see how many conversations you had with each person during the diary period. Keep in mind that this was only 14 days, so it does not reflect your entire social life, but it can give an indication.

Kwaliteit van interacties per partner

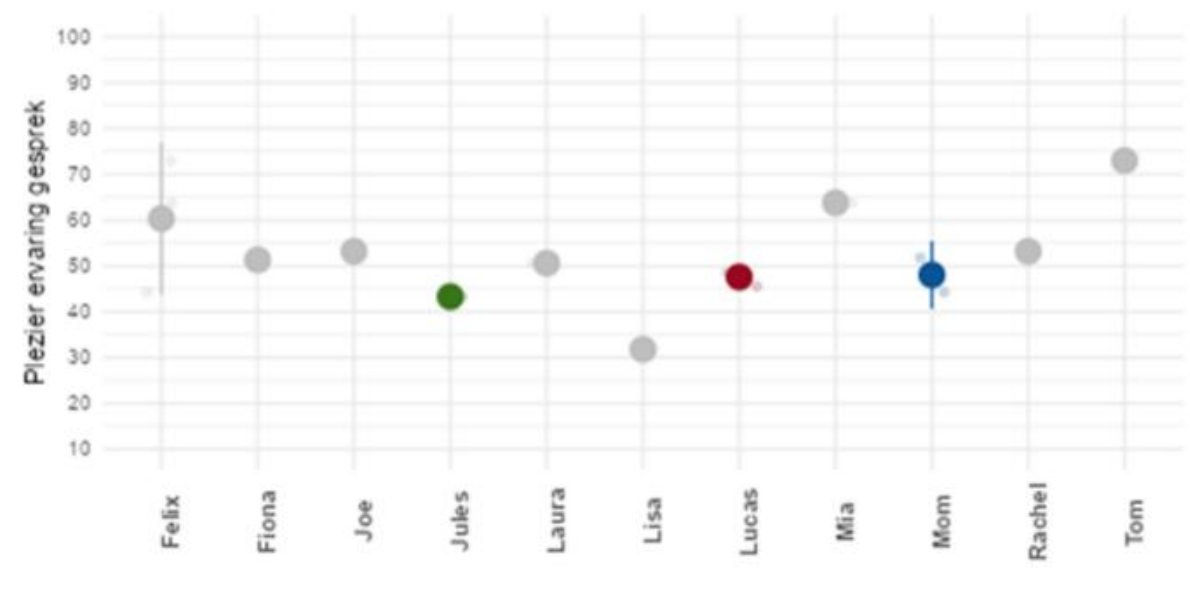

Explanation: Here you can see how you experienced the conversations with each person. The large dots summarize all conversations with one person, while the smaller dots around them represent each individual conversation. It's possible that you only had one conversation with some people during the 14 days — in that case, the "summary" reflects just that single interaction. The higher a dot is positioned, the more positively you experienced the interaction.

Conversations in which you felt supported:

2	22-10-2024 12:35	Felix,Tom	Face-to-face	90	Follow up meeting about the work project with another colleague - the project is almost done
11	26-10-2024 09:22	Mom	Telefoongesprek	30	She advised me on how to handle a difficult situation at work

Conversations about difficult topics:

8	24-10-2024 17:47	Lucas	Telefoongesprek	10	I did not feel so well and wanted to talk about that with him. He was there for me.
12	27-10-2024 11:25	Lucas	Face-to-face	60	We had a big disagreement about our upcoming holiday plans

Explanation: These are specific conversations that stood out during your diary period. Some of these might be useful to discuss with your therapist. You could talk about the topics of the conversation, or about how supported (or not) you felt.

Gesprekken en stemming / klachten

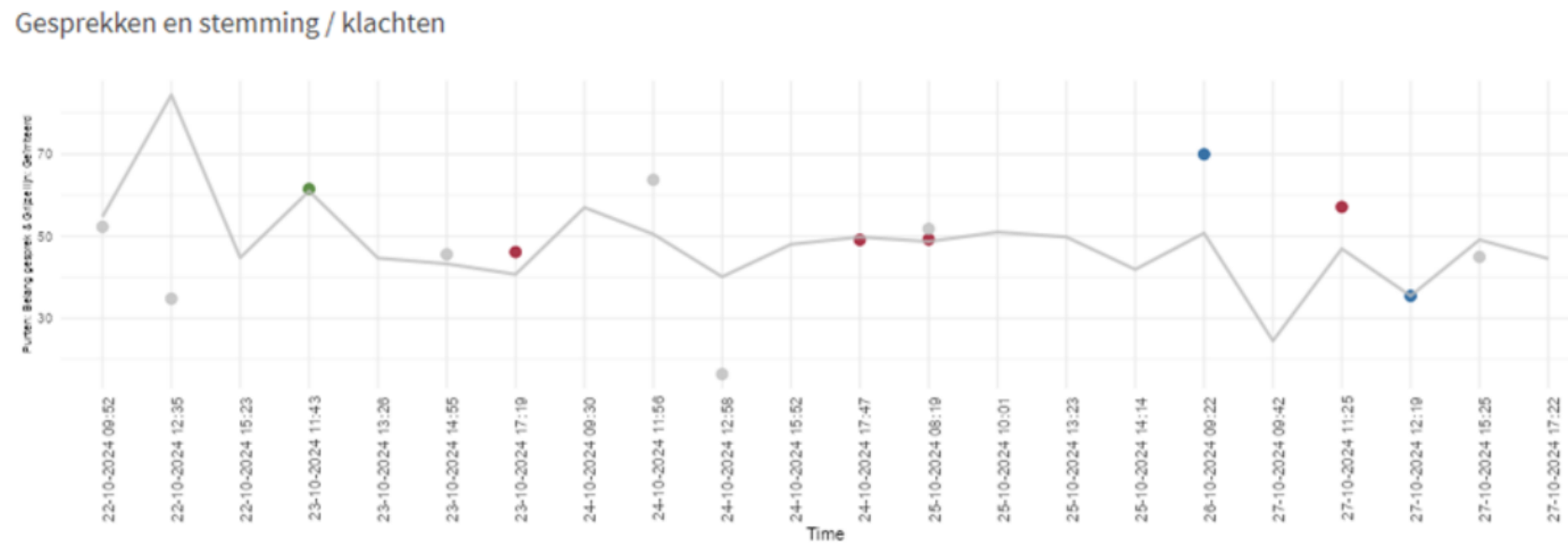

Explanation: This graph shows how irritated you felt during the 14 days of the diary (the grey line) and how supported you felt during your conversations (the dots). Each dot represents one conversation. The higher the dot, the more supported you felt. The higher the line, the more irritated you felt. Take note of the dates at the bottom of the chart. It's possible that you missed some diary entries, so this graph may not provide the complete picture.

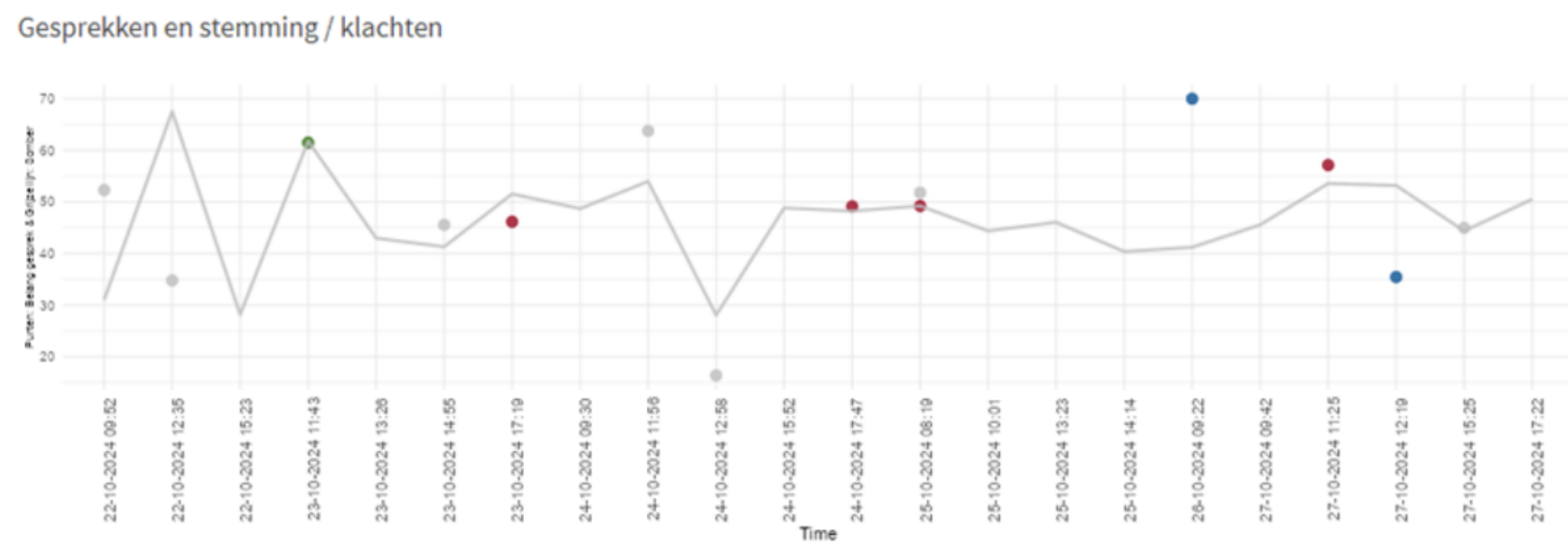

Explanation: Here is the same graph, but with a different diary question shown as the grey line: "At this moment I feel down."

Note. The example report contains fictional data with translated explanation texts and serves merely as an illustration of the provided feedback.

S.6 Protocol of the Evaluation Interviews (Original Dutch Version)

a. Patiënt

Vul deze vragen samen met de patiënt in voordat je de opname start.

“Eerst wil ik u vragen om een paar algemene vragen over u en uw behandeling in te vullen. Deze informatie zal ons later helpen om de groep patiënten te beschrijven die we in ons onderzoek hebben opgenomen. In elk wetenschappelijk artikel dat we schrijven, zullen we ervoor zorgen dat u niet persoonlijk identificeerbaar bent. Als er een vraag is die u niet wilt beantwoorden, dan kan dat natuurlijk altijd - dan slaan we die vraag gewoon over.”

1. Wat is uw geboortejaar?	
2. Wat is uw geslacht?	
3. Hoe is uw persoonlijke situatie?	o Alleenstaand<br>o Gehuwd of geregistreerd partnerschap<br>o Samenwonend<br>o Wel partner maar niet samenwonend<br>o Weduwe / weduwnaar<br>o Gescheiden<br>o Anders, namelijk: ________________
4. Wat is de hoogste opleiding die u heeft voltooid?	o Geen<br>o Lager algemeen onderwijs / basisonderwijs, of een gedeelte hiervan<br>o Lager beroepsonderwijs (lbo, vbo)<br>o Middelbaar algemeen onderwijs (mavo, mulo, ulo)<br>o Middelbaar beroepsonderwijs (mbo, mts)<br>o Hoger algemeen onderwijs (havo, vwo, hbs, gymnasium)<br>o Hoger beroepsonderwijs (hbo, hts)<br>o Wetenschappelijk onderwijs (universiteit)<br>o Anders, namelijk:
5.Wat is op dit moment uw belangrijkste dagelijkse bezigheid? (graag één antwoord aankruisen)	o Ik verricht betaald werk<br>o Ik zoek werk na verlies van mijn vorige baan / ben werkloos<br>o Ik ben gepensioneerd / met vervroegd pensioen (VUT)<br>o Ik ben (gedeeltelijk) arbeidsongeschikt<br>o Ik doe vrijwilligerswerk

	o Ik doe iets anders, namelijk__________________
6. Wat was de voornaamste reden dat u in behandeling bent (geweest) voor psychische klachten? Indien u een diagnose hebt gekregen, vul die hier dan in.	
7. Wat voor behandeling heeft u gehad? *Meerdere antwoorden mogelijk.*	o Psychologische behandeling<br>o Behandeling met medicatie<br>o Blended of online behandeling of e-health
8. Hoe lang bent u in totaal ongeveer in behandeling (geweest)?	

“Nu ben ik benieuwd naar uw ervaringen tijdens dit onderzoek. Ik zal u een paar open vragen stellen. We zijn erg geïnteresseerd in uw eerlijke mening en feedback - de positieve en de negatieve. Aarzel niet om om dingen te zeggen die u niet leuk vond, want we willen de procedure graag verbeteren voor toekomstig onderzoek. Dit deel van het interview willen we graag opnemen. Niemand behalve ik en Shanice Wortel zal naar deze opname beluisteren. Shanice en ik zullen de opname transcriberen en alle informatie die u zou kunnen identificeren verwijderen. Daarna wordt de opname verwijderd. We zullen de geanonimiseerde transcripties gebruiken voor onze analyse.”

Als de patiënt akkoord is, start nu de opname.

- Waarom heeft u ervoor gekozen om deel te nemen aan dit onderzoek?
- Wat wilt u uit dit onderzoek halen voor uw behandeling? Is dit gelukt?

“Nu zoomen we in op specifieke onderdelen van het onderzoek. Ik zal een aantal open vragen stellen over elk onderdeel.”

- **Het gesprek met je behandelaar en de onderzoeker om het dagboek te maken.**
    - Hoe heeft u dit deel van het onderzoek ervaren?
    - Wat vond u leuk aan dit deel? Wat vond u niet leuk?
    - Was alles duidelijk voor u?
    - Is er iets dat we kunnen verbeteren aan dit onderdeel?

- **Het netwerk interview met de onderzoeker.**
    - Hoe heeft u dit deel van het onderzoek ervaren?

- Wat vond u leuk aan dit deel? Wat vond u niet leuk?
    - Was alles duidelijk voor u?
    - Is er iets dat we kunnen verbeteren aan dit onderdeel?
    - Heeft u iets over uzelf / uw klachten geleerd tijdens het netwerk interview?
        - Zo ja, wat?
        - Zo niet, wat kunnen we verbeteren om het nuttiger te maken?

- **Het invullen van het dagboek.**
    - Hoe heeft u dit deel van het onderzoek ervaren?
    - Wat vond u leuk aan dit deel? Wat vond u niet leuk?
    - Was alles duidelijk voor u?
    - Is er iets dat we kunnen verbeteren aan dit onderdeel?
    - Zijn er problemen geweest tijdens de dagboek periode (bijv. technische problemen of problemen met het invullen van het dagboek tijdens het dagelijks leven)?
    - Heeft u iets over uzelf / uw klachten geleerd tijdens het dagboek?
        - Zo ja, wat?
        - Zo niet, wat kunnen we verbeteren om het nuttiger te maken?

- **De feedbacksessie met je behandelaar en de onderzoeker.**
    - Hoe heeft u dit deel van het onderzoek ervaren?
    - Wat vond u leuk aan dit deel? Wat vond u niet leuk?
    - Was de uitleg van de onderzoeker duidelijk voor u?
    - Waren de feedback grafieken duidelijk voor u?
    - Is er iets dat we kunnen verbeteren aan dit onderdeel?
    - Heeft u iets gemist in het feedbackrapport?
    - Heeft u iets over uzelf / uw klachten geleerd tijdens de feedbacksessie?
        - Zo ja, wat?
        - Zo niet, wat kunnen we verbeteren om het nuttiger te maken?
    - Denkt u dat uw therapeut iets nieuws over u / uw klachten heeft geleerd tijdens de feedbacksessie?
        - Zo ja, wat?
        - Zo niet, wat kunnen we verbeteren om het nuttiger te maken?

- Had u over het algemeen het gevoel dat dit onderzoek iets toevoegde aan uw therapie? Zo ja, wat was voor u de toegevoegde waarde?
- Hoe nuttig vond u dit onderzoek in het algemeen (op een schaal van 1 tot 10) voor uw therapie?

1	2	3	4	5	6	7	8	9	10

- Is er nog iets dat u wilt toevoegen?

b. Behandelaar

“Ik ben benieuwd naar uw ervaringen tijdens dit onderzoek. Ik zal u een paar open vragen stellen. We zijn erg geïnteresseerd in uw eerlijke mening en feedback - de positieve en de negatieve. Aarzel niet om dingen te zeggen die u niet leuk vond, want we willen de procedure graag verbeteren voor toekomstig onderzoek. Dit deel van het interview willen we graag opnemen. Niemand behalve ik en Shanice Wortel zal naar deze opname luisteren. Shanice en ik zullen de opname transcriberen en alle informatie die u of uw patiënt zou kunnen identificeren verwijderen. Daarna wordt de opname verwijderd. We zullen de geanonimiseerde transcripties gebruiken voor onze analyse.”

Als de behandelaar akkoord is, start nu de opname.

- Waarom heeft u ervoor gekozen om deel te nemen aan dit onderzoek?
    - Waarom specifiek met deze patiënt?
- Wat wilde u uit dit onderzoek halen voor de behandeling van deze patiënt? Is dit gelukt?
- Wat heeft u geleerd over deze patiënt door dit onderzoek?
- Wat waren uw behandeldoelen voor deze patiënt?
    - Heeft dit onderzoek geholpen om deze doelen te bereiken

“Nu zoomen we in op specifieke onderdelen van het onderzoek. Ik zal een aantal open vragen stellen over elk onderdeel.”

- **Het gesprek met de patiënt en de onderzoeker om het dagboek te maken.**
    - Hoe heeft u dit deel van het onderzoek ervaren?
    - Wat vond u leuk aan dit deel? Wat vond u niet leuk?
    - Was alles duidelijk voor u?
    - Is er iets dat we kunnen verbeteren aan dit onderdeel?
    - Heeft u iets over uw patiënt geleerd tijdens het maken van het dagboek?
        - Zo ja, wat?
        - Zo niet, wat kunnen we verbeteren om het nuttiger te maken?

- **De feedbacksessie met je behandelaar en de onderzoeker.**
    - Hoe heeft u dit deel van het onderzoek ervaren?
    - Wat vond u leuk aan dit deel? Wat vond u niet leuk?
    - Was de uitleg van de onderzoeker duidelijk voor u?
    - Waren de feedback grafieken duidelijk voor u?
    - Is er iets dat we kunnen verbeteren aan dit onderdeel?
    - Heeft u iets gemist in het feedbackrapport?
    - Heeft u iets over uw patiënt geleerd tijdens de feedbacksessie?
        - Zo ja, wat?
        - Zo niet, wat kunnen we verbeteren om het nuttiger te maken?

- Had u over het algemeen het gevoel dat dit onderzoek iets toevoegde aan de therapie voor deze patiënt? Zo ja, wat was voor u de toegevoegde waarde?
- Hoe nuttig vond u dit onderzoek in het algemeen (op een schaal van 1 tot 10) voor de therapie van deze patiënt?

1	2	3	4	5	6	7	8	9	10

“Nu wil ik verder uitzoomen en het hebben over het mogelijke gebruik van deze methode in het algemeen, los van het geval van deze specifieke casus.”

- Zou u deze methode voor andere patiënten gebruiken?
    - Waarom wel/niet?
- Voor welke patiëntengroepen denkt u dat deze methode bijzonder nuttig zou zijn en waarom?
- Voor welke patiëntengroepen zou deze methode <u>niet</u> geschikt zijn?
- During which kinds of therapy would you consider this method useful (e.g. CBT, IPT) and why?
- In welk deel van de therapie zou u deze methode nuttig vinden (bijvoorbeeld casusconceptualisatie, tijdens de behandeling, uitkomst monitoring) en waarom?

- Hoe ziet u de integratie van deze methode in de dagelijkse klinische praktijk?
    - Wat zou je nodig hebben om het gebruik van deze methode haalbaar te maken?

- Heeft u nog andere feedback of suggesties voor verbetering?

S.6 Protocol of the Evaluation Interviews (Translated English Version)

Patient

Fill in these questions together with the patient before starting the recording.

“First, I would like to ask you to answer a few general questions about yourself and your treatment. This information will help us later to describe the group of patients included in our study. In every scientific article we write, we will ensure that you cannot be personally identified. If there is a question you prefer not to answer, that is of course always possible — we will simply skip that question.”

1. What is your year of birth?	
2. What is your gender?	
3. What is your personal situation?	o single<br>o Married or registered partnership<br>o Living together<br>o In a relationship but not living together<br>o Widow / widower<br>o Divorced<br>o Other, namely: _________________
4. What is the highest level of education you have completed?	o None<br>o Primary education / elementary school, or part of it<br>o Lower vocational education<br>o Lower secondary education<br>o Intermediate vocational education<br>o Higher secondary education<br>o Higher professional education<br>o Academic education (university)<br>o Other, namely: _________________
5. What is currently your main daily activity? (please select one answer)	o I have paid employment<br>o I am looking for work after losing my previous job / I am unemployed<br>o I am retired / early retirement<br>o I am (partially) unable to work due to disability<br>o I do volunteer work<br>o Other, namely: _________________
6. What was the main reason you were (or have been) in	

treatment for psychological complaints?<br>If you received a diagnosis, please write it here.	
7. What kind of treatment did you receive? (Multiple answers possible)	o Psychological treatment<br>o Medication<br>o Blended or online treatment / e-health
8. Approximately how long have you been in treatment in total?	

“Now I am curious about your experiences during this study. I will ask you a few open questions. We are very interested in your honest opinion and feedback — both positive and negative. Please do not hesitate to mention things you did not like, as we would like to improve the procedure for future research. We would like to record this part of the interview. No one except Shanice Wortel and me will listen to this recording. Shanice and I will transcribe the recording and remove all information that could identify you. After that, the recording will be deleted. We will use the anonymized transcripts for our analysis.”

If the patient agrees, start the recording now.

- Why did you choose to participate in this study?
- What did you hope to gain from this study for your treatment? Did that happen?

“Now we will zoom in on specific parts of the study. I will ask some open questions about each part.”

The conversation with your therapist and the researcher to create the diary

- How did you experience this part of the study?
- What did you like about this part? What did you not like?
- Was everything clear to you?
- Is there anything we could improve about this part?

The network interview with the researcher.

- How did you experience this part of the study?
- What did you like about this part? What did you not like?
- Was everything clear to you?
- Is there anything we could improve about this part?

- Did you learn anything about yourself or your complaints during the network interview?
- If yes, what?
- If not, what could we improve to make it more useful?

Filling out the diary.

- How did you experience this part of the study?
- What did you like about this part? What did you not like?
- Was everything clear to you?
- Is there anything we could improve about this part?
- Were there any problems during the diary period (e.g., technical problems or difficulties completing the diary during daily life)?
- Did you learn anything about yourself or your complaints during the diary period?
    - If yes, what?
    - If not, what could we improve to make it more useful?

The feedback session with your therapist and the researcher.

- How did you experience this part of the study?
- What did you like about this part? What did you not like?
- Was the researcher's explanation clear to you?
- Were the feedback graphs clear to you?
- Is there anything we could improve about this part?
- Did you miss anything in the feedback report?
- Did you learn anything about yourself or your complaints during the feedback session?
    - If yes, what?
    - If not, what could we improve to make it more useful?
- Do you think your therapist learned anything new about you or your complaints during the feedback session?
    - If yes, what?
    - If not, what could we improve to make it more useful?

Overall evaluation:

Did you feel that this study added something to your therapy? If yes, what was the added value?

How useful did you find this study overall (on a scale from 1 to 10) for your therapy?

1	2	3	4	5	6	7	8	9	10

Is there anything else you would like to add?

Therapist

“I am curious about your experiences during this study. I will ask you several open questions. We are very interested in your honest opinion and feedback — both positive and negative. Please do not hesitate to mention things you did not like, because we want to improve the procedure for future research. We would like to record this part of the interview. No one except Shanice Wortel and me will listen to this recording. Shanice and I will transcribe the recording and remove all information that could identify you or your patient. After that, the recording will be deleted. We will use the anonymized transcripts for our analysis.”

If the therapist agrees, start the recording now.

- Why did you choose to participate in this study?
- Why specifically with this patient?
- What did you hope to gain from this study for the treatment of this patient? Did that happen?
- What did you learn about this patient through this study?
- What were your treatment goals for this patient?
- Did this study help to achieve these goals?

“Now we will zoom in on specific parts of the study. I will ask several open questions about each part.”

The conversation with the patient and the researcher to create the diary.

- How did you experience this part of the study?
- What did you like about this part? What did you not like?
- Was everything clear to you?
- Is there anything we could improve about this part?
- Did you learn anything about your patient while creating the diary?
    - If yes, what?
    - If not, what could we improve to make it more useful?

The feedback session with the patient and the researcher.

- How did you experience this part of the study?
- What did you like about this part? What did you not like?
- Was the researcher’s explanation clear to you?
- Were the feedback graphs clear to you?
- Is there anything we could improve about this part?
- Did you miss anything in the feedback report?
- Did you learn anything about your patient during the feedback session?
    - If yes, what?
    - If not, what could we improve to make it more useful?

Overall evaluation:

Did you feel that this study added something to the therapy for this patient? If yes, what was the added value?

How useful did you find this study overall (on a scale from 1 to 10) for this patient's therapy?

1	2	3	4	5	6	7	8	9	10

"Now I would like to zoom out and discuss the possible use of this method in general, separate from this specific case."

- Would you use this method for other patients?
- Why or why not?
- For which patient groups do you think this method would be particularly useful and why?
- For which patient groups would this method not be suitable?
- During which types of therapy would you consider this method useful (e.g., CBT, IPT) and why?
- At which stage of therapy would you find this method useful (for example case conceptualization, during treatment, outcome monitoring) and why?
- How do you see the integration of this method into daily clinical practice?
- What would you need to make the use of this method feasible?
- Do you have any other feedback or suggestions for improvement?

S.7 Research paradigm, Researcher Characteristics and Strategies to Enhance Trustworthiness

Qualitative approach and research paradigm

This study was guided by a pragmatic research paradigm, as we combined qualitative and quantitative methods to address an applied clinical question regarding the feasibility and utility of the intervention. Within this overall design, the primary qualitative strand was informed by an interpretivist approach, as we sought to understand how patients, therapists, and researchers experienced and made sense of the intervention within their specific clinical contexts. The quantitative strand served a supportive role by describing compliance and characteristics of the obtained ESM and PSN data. Together, this concurrent embedded mixed-methods multiple-case study design enabled us to integrate contextualized experiential accounts with descriptive quantitative indicators across cases.

Researcher characteristics and reflexivity

The research team comprised academic researchers from two Dutch universities and scientist-practitioners employed at the mental healthcare facility where data collection took place. MS initiated the project as a PhD candidate and completed it during subsequent employment as an assistant professor in methodology and statistics. MS carried out most of the data collection and analysis, supported by SW, who at the time was a research master's student working as a paid research assistant; both had specialized in clinical psychology during their master's training. The final author supervised the project and provided guidance and feedback. BM and JvH contributed as scientist-practitioners, combining clinical work with responsibility for innovation-oriented research at the mental healthcare facility where the data was collected. They facilitated access to participating therapists and patients. All authors considered patients' social context to be important in understanding and treating mental health problems and shared the view that this aspect is often insufficiently addressed in current clinical practice. MS and SW therefore remained attentive to how these assumptions may have shaped data collection and interpretation and discussed them throughout the study.

Techniques to enhance trustworthiness

To enhance trustworthiness, we drew on the four dimensions of credibility, transferability, dependability, and confirmability (Lincoln & Guba, 1985). Credibility was supported through triangulation across methods, data sources, and perspectives, including the views of patients, therapists, and researchers, as well as by including divergent cases such as dropout and low-compliance trajectories. Transferability was strengthened through detailed descriptions of the cases, clinical setting, and procedures, while dependability and confirmability were supported through prolonged engagement of both researchers in data collection, regular contact with scientist-practitioners and therapists, reflexive notetaking after each session, and transparent sharing of study materials. Due to the sensitive nature of the data, data summaries and completed framework matrices cannot be shared publicly but are securely stored by the research team as an audit trail of the analysis.

Reference: Lincoln, Y. S., & Guba, E. G. (1985). *Naturalistic inquiry*. Sage.
